# Amplification-Free Detection of MiRNA Signatures in Milk: A Noninvasive Sensing Approach for Early Diagnosis of Bovine Mastitis

**DOI:** 10.1021/acsmeasuresciau.6c00060

**Published:** 2026-06-17

**Authors:** Kandeel Shafique, Hedieh Haji-Hashemi, Łukasz Gontar, Ma̅ra Pilmane, Gergely Maróti, Beatriz Prieto-Simón

**Affiliations:** † Institute of Chemical Research of Catalonia, The Barcelona Institute of Science and Technology, Av. Països Catalans 16, 43007 Tarragona, Spain; ‡ Department of Electronic Engineering, Universitat Rovira i Virgili, 43007 Tarragona, Spain; § Research and Innovation Centre Pro-Akademia, 95-050 Konstantynów Łódzki, Poland; ∥ Institute of Anatomy and Anthropology, Rîga Stradin'š University, 9 Kronvalda Blvd., LV-1010 Rîga, Latvia; ⊥ SeqOmics Biotechnology Ltd., 6782 Morahalom, Hungary; # Institute of Plant Biology, HUN-REN Biological Research Center, 6726 Szeged, Hungary; ∇ ICREA, Pg. Lluís Companys 23, Barcelona 08010, Spain

**Keywords:** electrochemical DNA sensors, miRNA detection, milk analysis, bovine mastitis, early diagnosis

## Abstract

Bovine mastitis is an infectious disease with a significant impact on the dairy industry. It causes reduced milk yield and quality, and occasional mortality. Early and accurate diagnosis is challenging, being especially critical for subclinical mastitis lacking visible symptoms. MicroRNAs (miRNAs) are emerging as promising biomarkers for mastitis. Herein, using next-generation sequencing, a distinct miRNA signature (miRNA-146b, miRNA-221, miRNA-223) is identified, which is differentially expressed in healthy and naturally infected subclinical cows. Next, electrochemical DNA sensors are developed by self-assembling methylene blue-tagged oligonucleotides, complementary to each miRNA, on gold electrodes. MiRNA detection is achieved by monitoring hybridization-induced conformational changes using square-wave voltammetry. The sensors exhibit a sigmoidal response (dynamic range 0.1–100 nM), with a limit of detection of 0.1 nM and excellent selectivity. Importantly, they demonstrate strong diagnostic performance in milk-derived RNA extracts, with receiver operating characteristic–area under the curve values of 0.88, 0.80, and 0.70 for miRNA-221, miRNA-146b, and miRNA-223, respectively. Moreover, the responses are biologically meaningful, as correlation analysis highlights distinct associations of miRNA-146b and miRNA-221 with inflammation, and of miRNA-221 and miRNA-223 with microbial burden. The amplification-free detection of clinically relevant miRNAs in milk extracts represents a significant advancement toward noninvasive testing for the early diagnosis of mastitis. The development of simple, sensitive, and specific sensors targeting unique signatures of host immune-derived biomarkers promises a new framework to improve disease management and antimicrobial stewardship.

## Graphical abstract

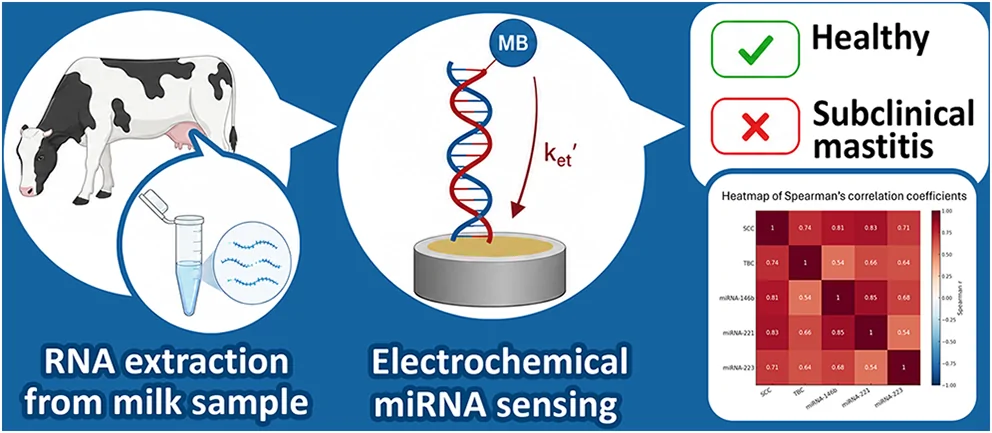

## 1 Introduction

The European dairy sector is a key contributor to the European Union’s agricultural economy. In 2023, the EU produced approximately 160.8 million tons of milk from more than 20 million cows, positioning itself as the world’s second-largest milk producer after India. Furthermore, the EU accounts for approximately 27.7% of global dairy exports, underscoring its pivotal role in the international dairy market.^1,2^ The sustainability of this sector depends on maintaining optimal health and welfare of dairy cows, with mastitis, leg disorders, and reproductive dysfunctions identified as the predominant health issues affecting productivity and animal well-being.^3,4^

Mastitis, defined as the inflammation of the mammary gland, is among the most prevalent and economically significant diseases in dairy cattle worldwide.^4,5^ It is primarily caused by intramammary bacterial infections. However, mycoplasmal, fungal and algal pathogens may also contribute to its etiology. Additionally, mechanical, thermal or chemical trauma can predispose animals to infection.^6,7^ Mastitis is clinically categorized into two forms: clinical and subclinical. Animals with clinical mastitis show visible symptoms such as milk abnormalities (presence of clots, flakes, watery consistency) and pronounced udder inflammation characterized by swelling, firmness and erythema. In contrast, subclinical mastitis lacks overt signs in milk or udder appearance, but it can progress to clinical mastitis if left untreated. Subclinical mastitis is more common, affecting up to 50% of dairy herds at any time. It poses diagnostic challenges due to the absence of any clinical indicators. It affects milk composition and quality, and its contagious nature serves as a persistent infection source, thus increasing intraherd transmission risk.^6,8^

Mastitis negatively affects health and welfare of dairy cows. It results in increased culling rates, premature drying off, and reduced milk yield and quality, the latter leading to loss of milk premiums.^6^ The disease also imposes substantial economic burdens through rise in veterinary and treatment costs, and increased labor requirements.^9^ Globally, mastitis is responsible for estimated annual economic losses of approximately USD 19.7 to 30 billion,^10^ with European losses of around Euro 3 billion each year.^11^ Mastitis also accounts for roughly 60–70% of antimicrobial usage in dairy farms, which promotes the emergence of antibiotic-resistant bacterial strains that compromise treatment efficacy and further complicate disease control.^10,12^ In this regard, early detection of mastitis, especially at the subclinical stage, is essential for effective management, reducing economic losses, and limiting disease transmission within herds, resulting in improved farm profitability and animal welfare.

Mastitis diagnosis faces several challenges, such as the absence of symptoms in subclinical or *Mycoplasma bovis*-induced cases.^13^ Laboratory methods like microbiological culturing and Polymerase Chain Reaction (PCR) are time-consuming, expensive, and not suitable for rapid on-farm testing. Culturing may yield false negatives due to low pathogen load or inappropriate media (e.g., *Mycoplasma spp*.), while PCR can miss infections due to inconsistent bacterial shedding or inability to distinguish viable from nonviable bacteria. Both methods are also prone to false positives: in culturing, this may arise from microbial contamination during sample handling, whereas in PCR, false positives can occur either from contamination with extraneous DNA or from detecting DNA of nonpathogenic microorganisms that are naturally present in milk but do not cause mastitis. Consequently, repeated testing is often necessary, further increasing costs.^8,14^ Immunoglobulin-based methods are also limited, as antibodies do not become detectable until weeks after infection. Indeed, none of these lab-based methods allows diagnosis at early infection stages, when animals are most infectious and treatment is most effective.^15^

On farm, diagnosis cannot rely on clinical symptoms, as they are delayed or absent in subclinical cases. Simpler methods like somatic cell count (SCC) or milk electrical conductivity are commonly used, but these are influenced by pathogen type and infection status.^16^ SCC EU cutoff is ≥200,000 cells/mL, but SSC is not always consistently elevated during infection (false negatives) and can also rise due to stress (false positives).^16,17^ Consequently, there is an urgent need for innovative, rapid and reliable diagnostic tools capable of overcoming these limitations, to facilitate early detection and effective management of bovine mastitis in field settings.

Recent diagnostic strategies are increasingly shifting from traditional pathogen detection toward evaluating host immune responses, which can provide earlier and more sensitive and accurate indicators of infection. Host-derived molecules such as nucleic acids, proteins and metabolites are released early during infection, making them promising biomarkers for rapid diagnosis. This approach overcomes limitations of pathogen-focused methods that often detect infection only after significant pathogen proliferation, thus delaying diagnosis, and just confirming pathogen presence rather than actual infection.^18^

In particular, microRNAs (miRNAs) have emerged as valuable biomarkers due to their stability in body fluids, organ specificity, and ability to reflect the host’s immune status. MiRNAs are small, noncoding RNA molecules that regulate gene expression post-transcriptionally, playing a crucial role in immune regulation and disease processes. In resting state, miRNAs maintain immune homeostasis by repressing pro-inflammatory genes, while during infection, changes in their expression can cause immune cell activation.^19^ miRNAs also participate in immune signaling networks, where they function in positive and negative feedback loops to fine-tune inflammation.^20^ Their expression patterns can provide valuable prognostic information, supporting early diagnosis and potentially predicting the course and severity of infections.^21^ Several miRNAs have been identified as biomarkers for mastitis in cows infected by various pathogens.^22^ However, most research has focused on induced mastitis models,^23–25^ often overlooking or underestimating the effects of mastitis acquired through natural pathogen exposure. Consequently, fewer studies have reported changes in milk miRNAs linked to naturally occurring mastitis.^22^ Notably, Ngo et al.^26^ emphasized a distinct miRNA signature associated with mastitis under natural infection conditions, highlighting the importance of investigating the disease in real-world settings.

Traditional miRNA detection techniques such as Northern blotting, RT-qPCR, microarrays, and next-generation sequencing (NGS), offer high sensitivity but are not suitable for point-of-care use due to their complexity and cost.^27,28^ In contrast, electrochemical biosensors have gained attention for miRNA detection because of their high sensitivity, rapid response, minimal sample preparation, and suitability for portable, on-site diagnostics. Their low cost, ease of operation, and compatibility with miniaturization make them promising tools for real-time, point-of-care applications, especially when integrated with mobile and cloud-based technologies.^29,30^ Electrochemical DNA sensors (E-DNA), in particular, show great potential for nucleic acid detection, including both DNA and RNA targets. These are reusable, operationally convenient, sequence-specific, and selective, making them a promising method for the detection of nucleic acids.^31^ These sensors rely on target-induced conformational changes, where hybridization of the electrode-bound DNA probe with its specific target alters the probe’s conformation, leading to a measurable change in the electrochemical signal. This hybridization guided by the base-pairing rules first described by Watson and Crick in 1953, enables the probe to selectively recognize and bind to its complementary nucleic acid strand. These sensors use a redox-labeled DNA capture probe, where one terminus is covalently modified with a redox reporter, commonly ferrocene (Fc) or methylene blue (MB), and the opposite terminus is functionalized with an alkanethiol to enable chemisorption onto the electrode surface. In the absence of the target, the redox reporter remains close to the electrode, allowing efficient electron transfer. Upon hybridization with a complementary target, the probe adopts a more rigid, double-stranded conformation that spatially separates the redox reporter from the electrode surface. This structural shift reduces electron transfer efficiency, resulting in a decrease in faradaic current which is proportional to the extent of hybridization.^31–33^

Considering the challenges associated with the early and accurate diagnosis of subclinical mastitis and leveraging the differential expression of miRNAs in healthy versus subclinical mastitis cases, here we demonstrate the development of three E-DNA sensors for the early stage detection of bovine mastitis. In this study, milk samples were classified as healthy or subclinical based on SCC values and microbiological analysis. Total RNA was extracted from those milk samples and subjected to NGS to identify miRNAs exhibiting consistent differential expression between healthy and subclinical mastitis samples. miRNA-146b, miRNA-221 and miRNA-223 were significantly upregulated in subclinical samples, supporting their utility as reliable biomarkers for early mastitis diagnosis. Next, we demonstrated the development of E-DNA sensors targeting the three selected miRNAs. Analytical parameters, including buffer composition and SWV frequency, were systematically optimized to maximize sensitivity and specificity. Following characterization in buffer, the sensors successfully detected the targeted miRNA signature in the total RNA extracted from milk in a cohort of 10 cows (comprising five healthy and five subclinical cases). This straightforward detection strategy represents a significant advancement toward rapid, sensitive, and noninvasive diagnosis of bovine mastitis, paramount for timely intervention and improved disease management.

## 2 Experimental Section/Methods

### 2.1 Materials

Sodium hydroxide (NaOH) was obtained from Cymit Quimica (Spain). Sulfuric acid (H_2_SO_4_) was purchased from PanReac AppliChem (Spain). Hydrogen peroxide 30% w/w (H_2_O_2_) was obtained from Scharlab (Spain). Phosphate-buffered saline (PBS) tablets, ethanol, magnesium chloride (MgCl_2_), and 6-mercapto-1-hexanol (6-MCH) were purchased from Sigma-Aldrich (Merck, Germany). Tris­(2-carboxyethyl)­phosphine hydrochloride (TCEP) was obtained from Thermo Fisher Scientific (Spain). The platinum counter and Ag/AgCl reference electrodes were obtained from Tianjin Aida Co. (China). Gold rod electrodes (2 mm diameter) were sourced from CH Instruments, Inc. (Austin, TX). Micro cloth pads, nylon polishing pads, and micropolish alumina powder (pore size: 1, 0.3 and 0.05 μm) were purchased from Redox.me (Sweden).

The LactoScan SCC analyzer, together with the manufacturer’s fluorescent staining reagents and disposable cuvettes, were obtained from Milkotester Ltd. (Bulgaria). Sterile 0.9% NaCl was purchased from POCH (Poland). Plate count agar was obtained from Biomaxima (Poland), and CHROMagar Mastitis medium was purchased from CHROMagar (France). Cellulose acetate syringe filters (0.8 μm) were obtained from Minisart, Sartorius (Germany). The exoRNeasy Maxi kit was obtained from QIAGEN (Germany). RNase-free 15 and 50 mL conical tubes were purchased from Googlab Scientific (Poland).

The Qubit RNA High Sensitivity (HS) Assay Kit was obtained from Thermo Fisher Scientific. The High Sensitivity RNA ScreenTape Assay Kit and TapeStation 4150 system were obtained from Agilent Technologies. The QIAseq miRNA Library Kit was obtained from QIAGEN (Germany).

TE buffer (10 mM Tris, 0.1 mM EDTA) and nuclease-free water (NF) were obtained from Integrated DNA Technologies, Inc. (Belgium).

Single stranded DNA (ssDNA) and miRNA sequences (Table S4) were synthesized and HPLC purified by Biosearch Technologies (UK). They were dissolved in nuclease-free water to prepare stock solutions at 100 μM concentration, aliquoted, and stored at −20 °C. RNA is particularly susceptible to degradation from heat, UV light and RNases, leading to diminished stability.^34^ Due to this inherent instability of RNA, which complicates synthesis and handling, increases costs, and necessitates stringent RNase-free conditions, we initially utilized ssDNA analogs of the target miRNA sequences for E-DNA sensors’ optimizations. Using ssDNA as a model target for sensing allows further effective translation to miRNA detection.^35^

Milli-Q water was used for the preparation of all buffers and reagent solutions, except for oligonucleotides. All reagents and materials were used as received unless otherwise stated.

### 2.2 Milk Sampling

Milk sampling was performed in two consecutive phases. During the initial screening phase, raw milk was collected from 35 lactating cows on the farm for three consecutive days, during the morning milking. Samples were taken separately from each of the four udder quarters into sterile RNase-free conical centrifuge tubes. The samples were kept under cool conditions in the laboratory prior to SSC determination. Following this three-day screening, cows were evaluated based on visual observations of the udder and SCC results. Ten animals were selected to represent two health statuses: healthy and subclinical mastitis. Specific udder quarters were identified for further sampling according to these criteria.

In the second sampling phase, conducted during the next morning milking session, larger volumes of milk (≥250 mL) per selected quarter were collected into sterile polypropylene containers. Prior to collection, the udder and teats were thoroughly cleaned, dried and disinfected. Foremilk was discarded, and samples were collected manually after machine milking. These aliquots were used for SCC determination, microbiological analysis, and RNA extraction.

### 2.3 Classification of Collected Milk Samples Based on SCC

Across each sampling round, we first performed a three-day screening of all lactating cows, collecting quarter milk during morning milking and determining SSC with a LactoScan SCC fluorescent image cytometer compliant with ISO 13366-1/IDF 18-1; each measurement comprised 16 technical replicates and the instrument’s working range was 10,000–10,000,000 cells/mL. Per cow, SCC classification was made using the average SCC of the screened quarter(s) over the 3 days, combined with visual assessment of udder health. Animals were assigned to health classes using the following a priori criteria: healthy – SCC < 200,000 cells/mL with no clinical signs; subclinical – SCC 200,000–500,000 cells/mL with no clinical signs. The screened quarter per selected animal was then used for downstream procedures (microbiology, RNA isolation), while detailed per-round SCC values and final sample assignments are provided in the Supporting Information (Tables S1, S2). For the electrochemical/NGS experiments, total RNA was extracted from the milk of cows classified as healthy and subclinical by the above SCC criteria.

### 2.4 Microbiological Analysis

For each selected udder quarter, conventional culture was performed immediately after milking on raw milk aliquots. TBC was quantified on Plate Count Agar following ISO 4833-2:2013:10-fold serial dilutions were prepared in 0.9% saline and plated using an automatic spiral plater (Easy Spiral, Interscience). Plates were incubated at 30 °C for 48 h and results reported as log_10_ CFU/mL. Presumptive identification of common mastitis pathogens was performed on CHROMagar Mastitis media (separate Gram-positive and Gram-negative) according to the manufacturer’s protocol (targeting *S. agalactiae*, *S. uberis*, *S. aureus*, *E. coli*, and collectively *Klebsiella/Enterobacter/Citrobacter* spp.): diluted samples were plated with the same instrument and incubated aerobically at 37 °C for 24 h before colony counting. All determinations were performed in duplicate. Due to limited recovery of some Gram-negative bacteria, downstream statistics focused on TBC and counts of *S. uberis* and *S. aureus* as predefined end points.

### 2.5 Extraction from Milk Samples for NGS and Electrochemical Analysis

Milk collected from the designated udder quarters was divided into matched fractions on the same sampling day: raw milk for SCC/microbiology and a parallel fraction preprocessed for RNA work to ensure all downstream assays originated from the identical time point and quarter.

Total RNA was then isolated following the exoRNeasy Maxi Kit protocol. Briefly, milk (50 mL) was centrifuged at 16,020*g*. The cream layer was removed, and the remaining sample was distributed into three 15 mL tubes and centrifuged again at 16,020*g*. From each tube, 2 mL of clear supernatant were recovered (6 mL total), which were then passed through a 0.8 μm cellulose acetate filter. Four mL of the filtrate were taken forward into the subsequent steps of the exoRNeasy Maxi Kit protocol. The extracted RNA was aliquoted, transported on dry ice, and stored at −80 °C. NGS analysis was conducted on RNA samples obtained from the first collection round to identify differentially expressed miRNAs, while electrochemical analysis was performed on RNA samples from the second collection round to detect the selected miRNA targets.

### 2.6 NGS Analysis of RNA Extracts

The total RNA amount for each sample isolated from milk was quantified using Qubit RNA High Sensitivity (HS) kit. The RNA qualities were assessed using an Agilent Tapestation 4150 system with the High Sensitivity RNA ScreenTape assay. Pooling was done as RNA yield from individual samples was not sufficient for reliable NGS analysis. Equimolar amounts of pooled total RNA samples were used for *in vitro* miRNA fragment library preparation (at least 10 individual total RNA samples were used for pool generation from healthy cows and those suffering subclinical mastitis). For miRNA library preparation the QIAseq miRNA Library Kit was used from QIAGEN by following the manufacturer’s instructions. The fragment libraries were sequenced on an Illumina NextSeq1000 platform, and at least 2 millions of paired end reads (2× 75 nt each) were generated for both pools. The reads were analyzed using QIAGen’s CLC Genomics Workbench software, and unique miRNA patterns were identified and described for the healthy and subclinical groups.

Here, it should be noted that pooling may mask interanimal variability. In future work, this limitation could be addressed by processing larger volumes of milk to obtain higher RNA yields from individual samples. However, RNA extraction kits are typically optimized for limited input volumes, and increasing the starting volume would require multiple parallel extraction steps, which would increase experimental complexity.

### 2.7 E-DNA Sensor Fabrication

Gold electrodes were used to prepare E-DNA sensors. First, electrodes were cleaned following a previously established protocol with slight modifications.^33^ Briefly, gold rod electrodes were polished in an aqueous suspension of alumina powder slurry, sequentially using 1 and 0.3 μm alumina on nylon pads for 2 min each, followed by 0.05 μm alumina on micro cloth pads for 5 min. To remove residual alumina particles, the electrodes were thoroughly washed with Milli-Q water, then sonicated in ethanol for 5 min and finally dried under a nitrogen stream.

Electrodes were then chemically cleaned by immersing them in piranha solution (a 1:3 mixture of 30% H_2_O_2_ and 98% H_2_SO_4_) for 10 min. Then the electrodes were thoroughly rinsed with Milli-Q water and dried under a stream of nitrogen. Next, electrodes were electrochemically pretreated using a three-electrode setup. First, the potential was cycled between −0.5 and 1 V versus Ag/AgCl at a scan rate of 0.1 V s^–1^ in an aqueous 0.5 M NaOH solution for 20 cycles. After rinsing with water, the electrodes were transferred to an aqueous 0.5 M H_2_SO_4_ solution and potential cycling was performed between −0.1 and 1.6 V versus Ag/AgCl at 0.1 V s^–1^ for 50 cycles.

Next, clean electrodes were modified with the selected capture probe. An aliquot of the thiolated DNA capture probe was thawed and subsequently reduced for 1 h at room temperature in the dark using a 1000-fold molar excess of TCEP. Thiolated DNA constructs are provided in their oxidized form which is not suitable for effective immobilization onto the gold surface, thus a disulfide reduction step is required to ensure their efficient immobilization on the gold electrodes. Following disulfide reduction, the solution was diluted to a final concentration of 1 μM in PBS (10 mM phosphate buffer, 137 mM NaCl, 2.7 mM KCl, pH 7.4) for subsequent surface functionalization.

For each miRNA target, a separate sensor was prepared by incubating 20 μL of the corresponding 1 μM specific capture probe solution onto electrochemically cleaned gold electrodes for 2 h in a humid chamber. Following this step, the electrodes were thoroughly rinsed with Milli-Q water before immersing them in a 3 mM 6-MCH solution prepared in PBS overnight at room temperature. The sensors were rinsed again with Milli-Q water. Finally, to equilibrate them, the sensors were immersed in PBS for 30 min before use.

### 2.8 Electrochemical Measurements and Acquisition of Signal Gain

All electrochemical measurements were conducted using a three-electrode setup comprising a gold working electrode, an Ag/AgCl reference electrode, and a platinum counter electrode, connected to an IviumStat potentiostat. All measurements were performed at room temperature.

SWV was used to measure the electrochemical response of the biosensor, with measurements performed by scanning potentials from −0.05 to −0.45 V versus Ag/AgCl, using a pulse amplitude of 0.025 V, a potential step of 0.003 V, and an optimized frequency of 50 Hz.

To evaluate the sensor’s response, the signal gain percentage was calculated using eq 1:

1 $$\mathrm{Signal}\,\;g\mathrm{ain}\%=\left(\left(I_{0}-I_{\mathrm{target}}\right)/I_{0}\right)\times 100$$

where *I*_0_ and *I*_target_ represent the current measured in the absence and in the presence of the target analyte, respectively.

### 2.9 Optimization of SWV Frequency and Buffer

To determine the SWV frequency providing the maximum signal gain for each biosensor, sensors were prepared with capture probes complementary to miRNA-146b, miRNA-221 and miRNA-223, and tested across 15 frequencies: 2, 5, 10, 20, 30, 40, 50, 60, 70, 80, 90, 100, 150, 175 and 200 Hz. We first recorded SWV measurements in PBS at each frequency to establish a stable baseline response. The sensors were then incubated for 1 h in a humid chamber with 100 nM of the complementary DNA target prepared in PBS, after which SWV measurements were recorded in the target solution across all frequencies. Those SWV measurements were used to calculate signal gain at each frequency.

To find the optimal hybridization conditions, electrodes were prepared with the capture probe specific to miRNA-223 and with a nonspecific capture probe (control), and evaluated their hybridization efficiency using target solutions prepared either in PBS (10 mM, pH 7.4) or Tris-HCl (10 mM, pH 7.4). First SWV measurements were recorded in the respective buffers to establish baseline responses, before incubating the sensors with 100 nM miRNA-223 DNA target for 1 h in a humid chamber. Following hybridization, SWV measurements were recorded in the corresponding target solutions.

### 2.10 Dose–Response Curves

To determine the analytical performance of the three E-DNA sensors prepared to detect miRNA-146b, miRNA-221 and miRNA-223, initially titration experiments were conducted using DNA targets corresponding to each miRNA. For each target, measurements were performed using three independently prepared sensors (*n* = 3). Prior to target incubation, background signals were recorded through SWV measurements in PBS at 5 min intervals until a stable response was obtained. The sensors were then sequentially incubated with increasing concentrations of the corresponding DNA target (0.01, 1, 10, 50, 100, and 1000 nM) in a humid chamber for 1 h per concentration. After each incubation, SWV measurements were performed in the respective target solutions, and the signal gain % was calculated across all concentrations.

The same procedure was applied to miRNA targets under RNase-free conditions. The recorded responses across all concentrations were then used to construct dose–response curves for both DNA and miRNA targets.

### 2.11 Electrochemical Analysis of Milk Samples Collected from Cows

To evaluate the efficiency of the developed and optimized sensors for diagnosing bovine mastitis through the analysis of the selected panel of miRNAs in milk, a total of 30 sensors were prepared, with separate sets functionalized with capture probes specific to miRNA-146b, miRNA-221 and miRNA-223. These sensors were used to detect their respective targets in RNA extracts obtained from milk samples collected from ten cows, five healthy and five suffering bovine mastitis at the subclinical stage.

Before analysis, aliquots of total RNA extracted from milk, stored at −80 °C, were thawed and diluted 1:1 in PBS. The developed sensors were first equilibrated in PBS for 30 min. Then SWV measurements were recorded in PBS at 5 min intervals at 50 Hz until a stable baseline was achieved. Next, 20 μL of the diluted RNA extracts were incubated on each sensor for 1 h in a humid chamber, followed by SWV measurements to evaluate sensor’s response. Each sample was tested for the presence of miRNA-146b, miRNA-221 and miRNA-223, using a separate sensor. The signal gain was calculated to determine hybridization efficiency. To evaluate the performance of all miRNA sensors toward the diagnosis of bovine mastitis, ROC curves were generated by plotting the true positive rate against the false positive rate at various signal thresholds. AUC values were then calculated to quantify the sensor’s diagnostic performance.

### 2.12 Statistical Analysis

Statistical analysis was performed using GraphPad Prism, employing an unpaired *t* test for all necessary comparisons. Correlation analysis was performed using Spearman’s rank correlation. A 95% confidence interval was maintained, and *p* < 0.05 was considered statistically significant. Detailed analyses for each group comparison are presented in the relevant sections.

## 3 Results and Discussion

### 3.1 SSC and Microbiological Characteristics of Milk Samples

Milk samples were collected from 35 dairy cows from a herd of 45 Holstein–Friesian animals in two sampling rounds. In the first round, milk samples were classified according to their SSC levels (see Table S1). Samples from five healthy cows and five cows with subclinical mastitis were selected for miRNA profiling via NGS to identify miRNAs differentially expressed in mastitic cows.

In the second round, milk was again sampled and SCC classified to select five healthy cows and five cows with subclinical mastitis (Table S2). RNA extracts were obtained from the milk of these selected animals, which were later analyzed using the developed electrochemical biosensors. Milk was also microbiologically analyzed (Table S3). Microbiological and SSC data were jointly evaluated to unveil links between etiology and host response, identify diagnostic limitations, and later confirm diagnostic accuracy of the sensing results. On the sampling day, SCC showed a clear stepwise separation between the two groups: the median SCC was 1.07 × 10^3^ cells/mL in healthy, and 271 × 10^3^ cells/mL in subclinical cows. The interquartile ranges (IQR) did not overlap across the groups, 0.0–3.40 × 10^3^ cells/mL in healthy and 96.7–523 × 10^3^ cells/mL in subclinical, which indicates distinct distributions rather than marginal shifts. The total bacterial count (TBC) followed the same pattern: median TBC was 2.43 log_10_ CFU/mL in healthy, and 3.34 log_10_ CFU/mL in subclinical. Here, the IQR for subclinical (3.30–3.37 log_10_ CFU/mL) was clearly higher than the IQR for healthy (2.30–2.73 log_10_ CFU/mL). Targeted culture determination assay identified *Streptococcus uberis* (*S. uberis*) in all examined cows (10/10; 100%). *Streptococcus agalactiae* (*S. agalactiae*) was absent in healthy (0/5; 0%), but prevalent in subclinical (5/5; 100%). *Staphylococcus aureus* (*S. aureus*) was detected in both categories: 3/5 healthy (60%) and 1/5 subclinical (20%). *Escherichia coli* (*E. coli*) was not detected in either healthy or subclinical cows (0/10; 0%). Finally, coisolation of two or more targeted pathogens (*S. uberis*, *S. agalactiae*, *S. aureus*, *E. coli*) occurred in 3/5 healthy (60%) and 5/5 subclinical (100%), consistent with increasing microbiological complexity alongside higher SCC.

The increasing levels of both SCC and TBC in milk from healthy to subclinical cases agree with the contemporary understanding of SCC as a reliable host-response readout of intramammary infection that reflects inflammation rather than culture load per se. Specialized reviews emphasize that SCC rises with neutrophil influx and separates health states at the population level, while its magnitude is modulated by pathogen type and cow-level factors. These observations support the use of SCC to discriminate groups even when TBC distributions partly overlap.^36,37^

The recovery of *S. uberis* across both groups in our data set is consistent with its ecology as a predominantly environmental mastitis pathogen with multiple reservoirs in the housing environment. Studies show that *S. uberis* genotypes circulate widely on farms and can be traced between environmental niches and udder infections, supporting a scenario of continual exposure and diverse clinical expression (from low-grade intramammary infection to overt disease) as observed here.^38–40^ By contrast, *S. agalactiae* concentrated in the subclinical group, aligning with its status as a contagious udder pathogen that often produces persistent, paucisymptomatic infections and elevates bulk-tank SCC unless actively controlled. Modern clinical guidance and summaries of control programs continue to classify *S. agalactiae* as primarily contagious, with transmission during milking and a strong subclinical footprint, which mirrors its distribution in our data.^41,42^ Recovery of *S. aureus* across both categories, including low-level detections in nominally healthy cows, is compatible with its well-documented propensity for chronic, biofilm-associated persistence and intermittent shedding. That biology explains sporadic low culture counts at single time points despite biologically relevant infection and underscores the value of interpreting culture together with a contemporaneous host-response marker such as SCC.^43–45^

Finally, the increase in codetection of ≥ 2 targeted pathogens from healthy to subclinical supports the growing view that bovine mastitis is frequently polymicrobial, with community interactions shaping inflammation and clinical expression. Integrative culture-plus-microbiome work indicates that mixed communities can intensify disease and blur one-pathogen/one-phenotype expectations, an interpretation that aligns with the coisolation patterns herein observed.^46,47^

In sum, these microbiology–SCC relationships mirror current epidemiology: ubiquitous environmental pressure from *S. uberis* across health states; a subclinical, contagious signal from *S. agalactiae*; and *S. aureus* spanning categories due to persistence. Interpreting SCC and culture as complementary signals (i.e., host response and etiologic landscape) provides a coherent framework for diagnosis and motivates the need of molecular-based methods of detection where subclinical discrimination is most challenging.

### 3.2 Identification of Differentially Expressed miRNAs

As described, in a first collection round, milk from ten selected cows (healthy *n* = 5; subclinical *n* = 5) was pooled and analyzed through NGS to identify miRNAs differentially expressed. A total of ∼4.25 and 3.82 million raw reads were generated for the subclinical and healthy samples, respectively. After Unique Molecular Identifiers correction and filtering, 851,171 high-quality reads were retained in the subclinical data set and 602,616 in the healthy data set. Both data sets showed an average quality score above Q35 which exceeded the Q30 threshold of >99.9% base call accuracy, confirming the reliability of sequencing data. Mapping against bovine miRBase and RNAcentral showed that ∼34% of reads in the subclinical sample (292,512 reads) and ∼25% of reads in the healthy sample (151,442 reads) could be annotated as known bovine miRNAs, providing sufficient sequencing depth and annotation coverage for differential expression analysis.

Attention was given to miRNAs that satisfied two criteria: (i) detectable baseline expression in healthy milk, which provides enough abundance for downstream biosensing applications, and (ii) consistent and statistically significant upregulation in subclinical mastitis, supporting diagnostic potential.

Based on these criteria, three miRNAs were selected as candidate biomarkers: miRNA-223, miRNA-221 and miRNA-146b. All three were present at baseline levels in healthy milk, and showed significant upregulation in subclinical mastitis. For example, miRNA-223 increased from less than 400 reads in healthy milk to 2,279 reads in the subclinical sample (∼62-fold change). Although miRNA-221 and miRNA-146b were not among the most abundant species, they demonstrated strong and statistically significant upregulation (Table 1) (detailed sequencing data sets are available via OSF).

**Table 1:** NGS-Based Differential Expression of the Selected miRNAs

miRNA	fold change	log2FC	expression	*p*-value
miRNA-223	62.40	5.96	upregulated	<0.001
miRNA-221	30.57	4.93	upregulated	<0.001
miRNA-146b	2.79	1.48	upregulated	<0.001

These findings demonstrate that miRNA-223, miRNA-221 and miRNA-146b are upregulated in the milk of cows suffering subclinical mastitis. Importantly, all three miRNAs were present at measurable baseline levels in healthy samples. While several highly abundant miRNAs dominated the sequencing profiles, they did not exhibit large fold changes between groups. This highlights the diagnostic value of moderately expressed but differentially regulated miRNAs, such as the three selected miRNAs, as more reliable biomarkers for subclinical mastitis detection.

The upregulation of these miRNAs in bovine mastitis is further supported by previous literature. MiRNA-223 has been consistently shown to increase in milk from naturally infected cows^22^ and in bovine mammary epithelial cells stimulated with pathogens or lipopolysaccharides.^48^ MiRNA-146b was reported as differentially expressed in transcriptome studies of mastitis-infected dairy cows,^49^ and miRNA-221 has been found upregulated in bovine mammary gland tissue and exosomes during mastitis.^50^ Most prior studies involved induced mastitis models or *in vitro* cell culture systems, whereas the present work confirms the relevance of the selected miRNAs in milk collected from cows naturally infected with subclinical mastitis.

### 3.3 E-DNA Sensors for miRNA-146b, miRNA-221 and miRNA-223 Detection

We developed and optimized reagentless, single-step E-DNA sensors designed for the detection of the selected panel of miRNAs based on a conformational change-based mechanism. Each sensor consists of a ssDNA capture probe covalently immobilized on a gold electrode surface, and modified with a methylene blue redox reporter at the distal end. Upon hybridization with a complementary nucleic acid, the probe undergoes a conformational change that alters the electron transfer between the redox reporter and the electrode surface, resulting in a measurable electrochemical signal. This mechanism facilitates rapid, sensitive and specific detection of nucleic acids, without the need for additional reagents. Moreover, the sensor has the possibility of simple regeneration.^32,51^

We designed three E-DNA sensors targeting miRNA-146, miRNA-221 and miRNA-223. Sensors were initially optimized using synthetic DNA analogs of the target miRNAs, focusing on key parameters, including the frequency used for SWV measurements and the hybridization buffer. After establishing optimal experimental conditions, we tested the analytical performance of the sensors toward their response to DNA targets spiked in buffer, determining selectivity and generating the corresponding titration curves. Following this, we transitioned to the miRNA targets and generated titration curves for the three selected miRNAs.

#### 3.3.1 Frequency Optimization

The three designed E-DNA sensors rely on hybridization-induced changes in electron transfer at the electrode surface. The signal gain, defined as the change in response upon target hybridization, is influenced by the frequency of the applied square-wave potential pulses during interrogation. Indeed, the frequency of the interrogating SWV is a critical parameter in electrochemical sensing, as it directly influences electrode reaction kinetics which can impact the signaling of electrochemical sensors.^52,53^ High frequencies emphasize fast electron transfer events, being more sensitive to rapid redox reactions, while low frequencies highlight slow processes.^53^ The optimal frequency differs depending on probe structure, characteristics of redox reporter, and other sensor design elements, such as the nature of the coating monolayer and the probe density on the electrode surface.^32^

The first step in sensor design was the selection of the SWV frequency providing the highest and most reproducible signal gain, along with clear and well-resolved voltammograms. For this purpose, SWV frequencies within the range from 2 to 200 Hz were swept both in PBS and in a 100 nM solution of each DNA target (analog of miRNA-146b, miRNA-221 or miRNA-223) prepared in PBS, and signal gain at each frequency was calculated. As shown in Figure 1 (signal gain at 2 Hz not included to facilitate comparison), both the magnitude and direction of signal gain varied across different frequencies. These results agree with previous works that report the precise control over the magnitude and direction of the signal by tuning the SWV frequency. This tunability enables the design of both ″signal ON″ and ″signal OFF″ sensors,^54^ as corroborated by our findings. At low frequencies, such as 2 Hz, all three DNA targets exhibited "signal ON" responses. However, as the frequency increased to medium and high ranges (20 Hz and above), the signal gain transitioned to "signal OFF". Despite variations among individual DNA targets, a stable and reproducible response was observed at 50 Hz for all three sensors. For the DNA analog of miRNA-146b, the signal gain was exceptionally high, 2,958% ± 222 at 2 Hz, which then decreased to 87% ± 13.7 at 5 Hz. Beyond this point, the signal direction reversed, transitioning to "signal OFF", and at 50 Hz, a reproducible signal gain of 70% ± 0.55 was observed. Similarly, the DNA analog of miRNA-221 showed a “signal ON” of 135% ± 24.5 at 2 Hz, which gradually declined with increasing frequency and showed a “signal OFF” of 70% ± 1.39 at 50 Hz. In the case of the DNA analog of miRNA-223, a “signal ON” response of 453% ± 44.5 was observed at 2 Hz, followed by a gradual decline at 5 and 10 Hz, transitioning to "signal OFF” at higher frequencies, with 57% ± 2.4 signal at 50 Hz.

**Figure 1:**
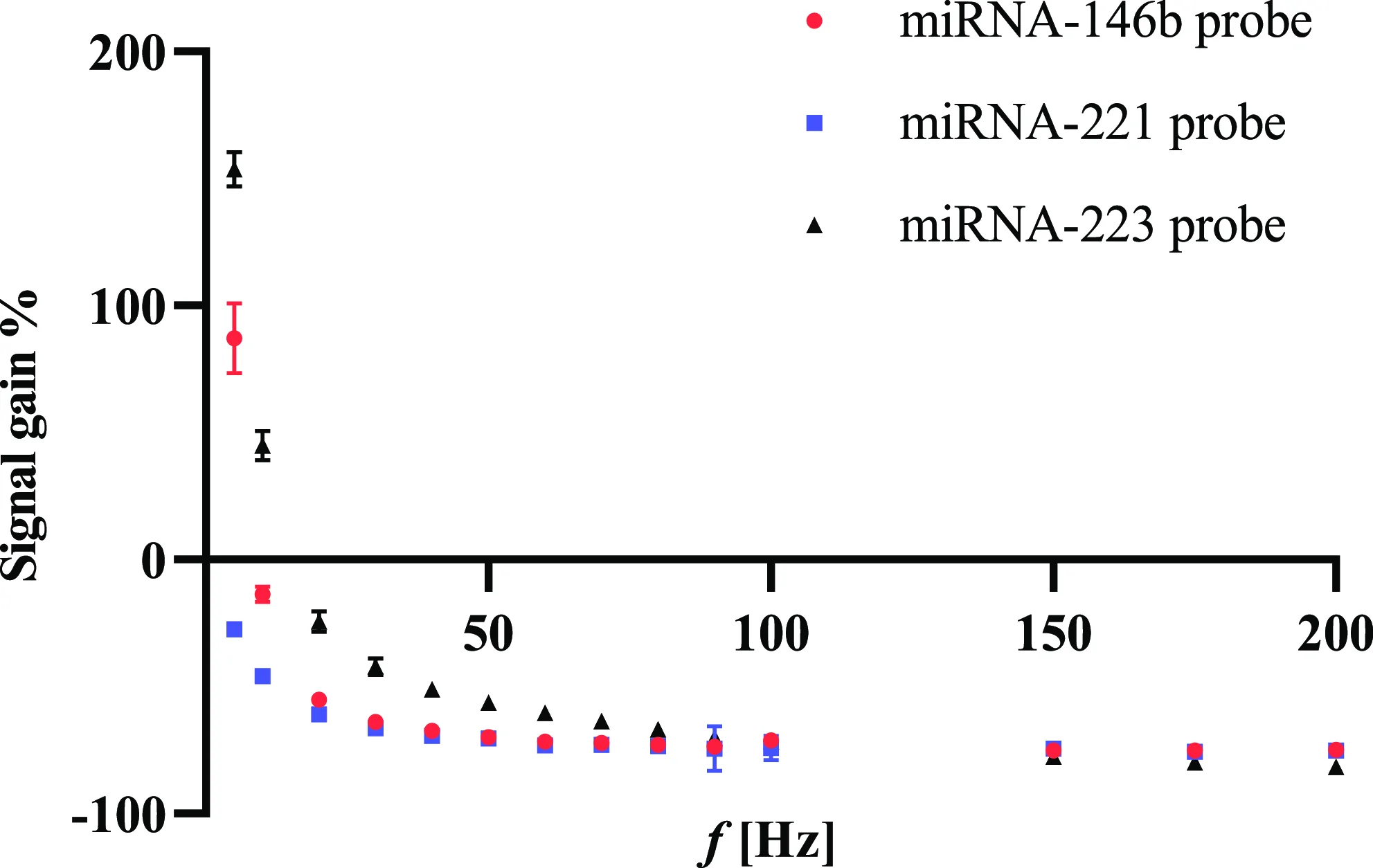
Frequency sweep analysis for E-DNA sensors designed to detect DNA analogs of miRNA-146b, miRNA-221 and miRNA-223. Signal gain was measured across a range of frequencies before and after incubation with a 100 nM solution of each DNA target. Values represent the average signal gain obtained from three independent sensors, with error bars indicating the standard deviation.

Among all tested frequencies, 50 Hz provided the best overall signal quality, showing stable signal gain with minimal variation between measurements, whereas higher frequencies showed some increase in gain, but with reduced signal quality due to noise. Meanwhile, 2 Hz consistently produced a strong "signal ON" response but showed poor reproducibility.

The observed high signal gains at low frequencies (e.g., 2 Hz) arise from the different electron transfer kinetics of the bound and unbound states. The unbound probe, which exhibits faster electron transfer, contributes less to the measured current under these conditions, while the target-bound probe, characterized by slower electron transfer, retains a relatively larger contribution. As a result, the baseline associated with the unbound state is reduced, leading to a smaller denominator in the signal gain calculation and, consequently, an apparent amplification of the signal.^55^ In parallel, at low frequencies background Faradaic processes, including oxygen reduction, contribute to the measured current and introduce fluctuations in the baseline^56^ thus increasing sensor-to-sensor variability. Together, these effects explain why low frequencies yield high apparent signal gains but poorer reproducibility. Conversely, at high frequencies, the noise caused by the electrochemical analyzer increases, affecting the signal-to-noise ratio.^33^

Given the limitation of sensor-to-sensor variability at 2 Hz and low signal-to-noise ratio at high frequencies, and to maintain consistency across all three targets, we chose to conduct further experiments at 50 Hz.

#### 3.3.2 Selectivity and Specificity Assessment

After selecting a working frequency of 50 Hz, and PBS as buffer (see SI, Section 2, Figure S1), we evaluated the selectivity of the three E-DNA sensors developed. Each sensor was challenged with four types of DNA targets at 100 nM: a fully complementary DNA sequence, two noncomplementary DNA sequences, a cocktail containing equal concentrations of all three DNA targets and a two-base mismatched (2bpmm) DNA sequence.

As shown in Figure 2, the miRNA-146b sensor responded strongly to its complementary target, with a signal gain of ∼71%, which was much higher than ∼7% response to the noncomplementary sequences. When tested with the 2bpmm sequence, the response dropped to ∼46%, indicating some recognition but less than the fully matched sequence. In the cocktail mixture, the sensor still showed a high signal gain of 72%, demonstrating that it can selectively detect its target even in complex mixtures.

**Figure 2:**
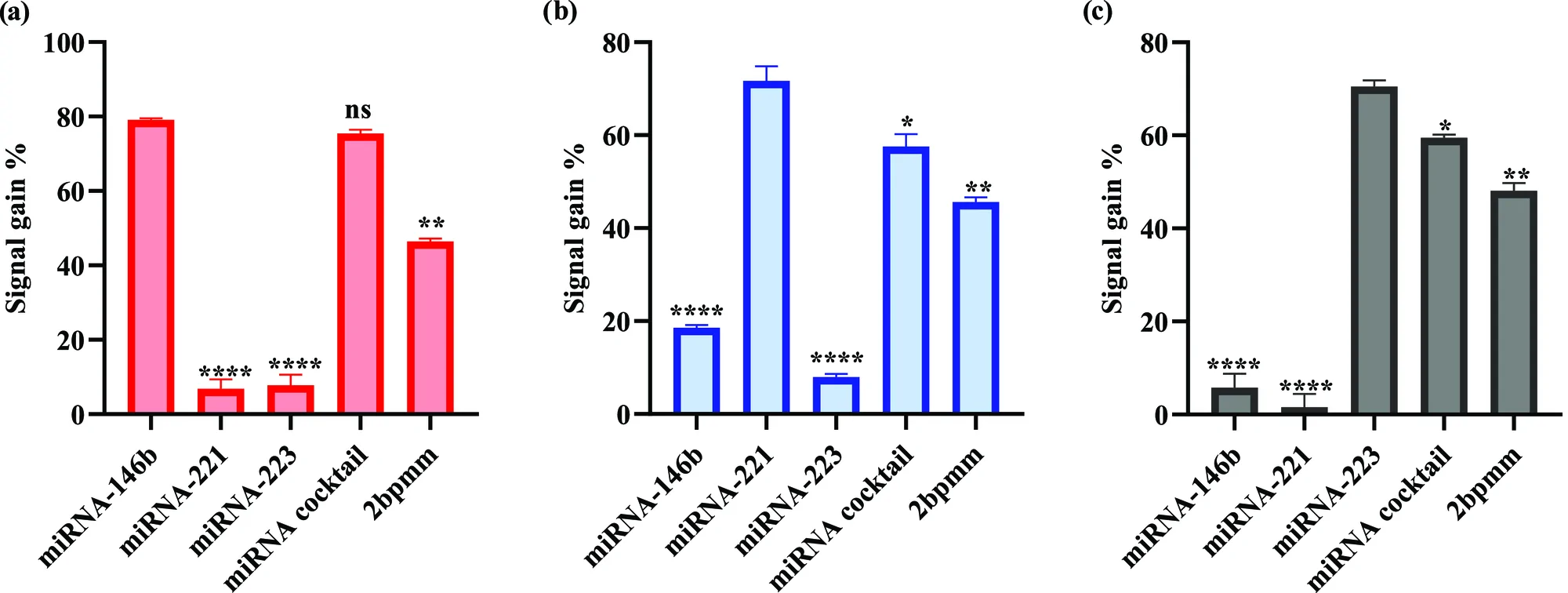
Selectivity evaluation of the E-DNA sensors designed to detect miRNA-146b, miRNA-221 and miRNA-223 (a–c). Each type of sensor was tested with 100 nM solutions of the fully complementary DNA target corresponding to the respective miRNAs, two noncomplementary DNA sequences, a mixture of all three DNA targets, and a 2bpmm DNA sequence, all prepared in PBS buffer. Values represent the average signal gain obtained from three independent sensors for each test group, with error bars indicating the standard deviation. Statistical significance was determined using an unpaired *t* test, with *p* < 0.0001 (****), *p* < 0.01 (**) and *p* ≥ 0.01 (*) showing significant, and ns denoting no significant difference.

Similarly, the miRNA-221 sensor showed a signal gain of ∼72% to its complementary target, significantly higher than 18 and 8% to noncomplementary sequences. The sensor showed a signal gain of ∼46% to the 2bpmm sequence, and ∼56% in cocktail. The reduced response in the cocktail can be attributed to competition among sequences for hybridization sites, a common phenomenon in multicomponent nucleic acid mixtures that can transiently hinder hybridization efficiency.^57^ Despite this, the sensor maintained good selectivity, indicating its potential to recognize its target in the presence of other sequences.

The miRNA-223 sensor also responded strongly, with signal gain of ∼70%, to its fully complementary target, with much lower responses (6 and 2%) to noncomplementary sequences. When challenged with the 2bpmm sequence, it showed signal gain of ∼48%, and in the cocktail, sensor showed ∼60% signal gain, although lower than the perfect match, this value still indicates good selectivity.

These results confirm that all three sensors are highly selective, effectively distinguishing their specific targets from similar or nontarget sequences even in complex mixtures. The reduced response to mismatched sequences is likely due to less stable DNA duplex formation caused by mismatches, which decreases hybridization efficiency.^58^ This high selectivity is essential for accurately detecting targets in real biological samples.

#### 3.3.3 Analytical Performance of E-DNA Sensors toward the Detection of DNA and RNA Analogs

To compare the performance of E-DNA sensors toward the detection of DNA and RNA targets, titration curves were plotted in PBS. E-DNA sensors specific to miRNA-146b, miRNA-221 and miRNA-223 were prepared. Titration curves were obtained using DNA target sequences corresponding to each miRNA, and the three target miRNAs, all prepared in PBS within a concentration range from 0.01 to 1000 nM. The observed signal gain obtained using the optimal conditions exhibited a sigmoidal trend for all three DNA targets and miRNA targets, indicative of hybridization saturation as the target concentration increased (Figure 3). The limit of detection (LOD), determined as the concentration corresponding to 10% of the maximum signal in the saturation region, was estimated to be approximately 0.1 nM in buffer for all sensors.^59^

**Figure 3:**
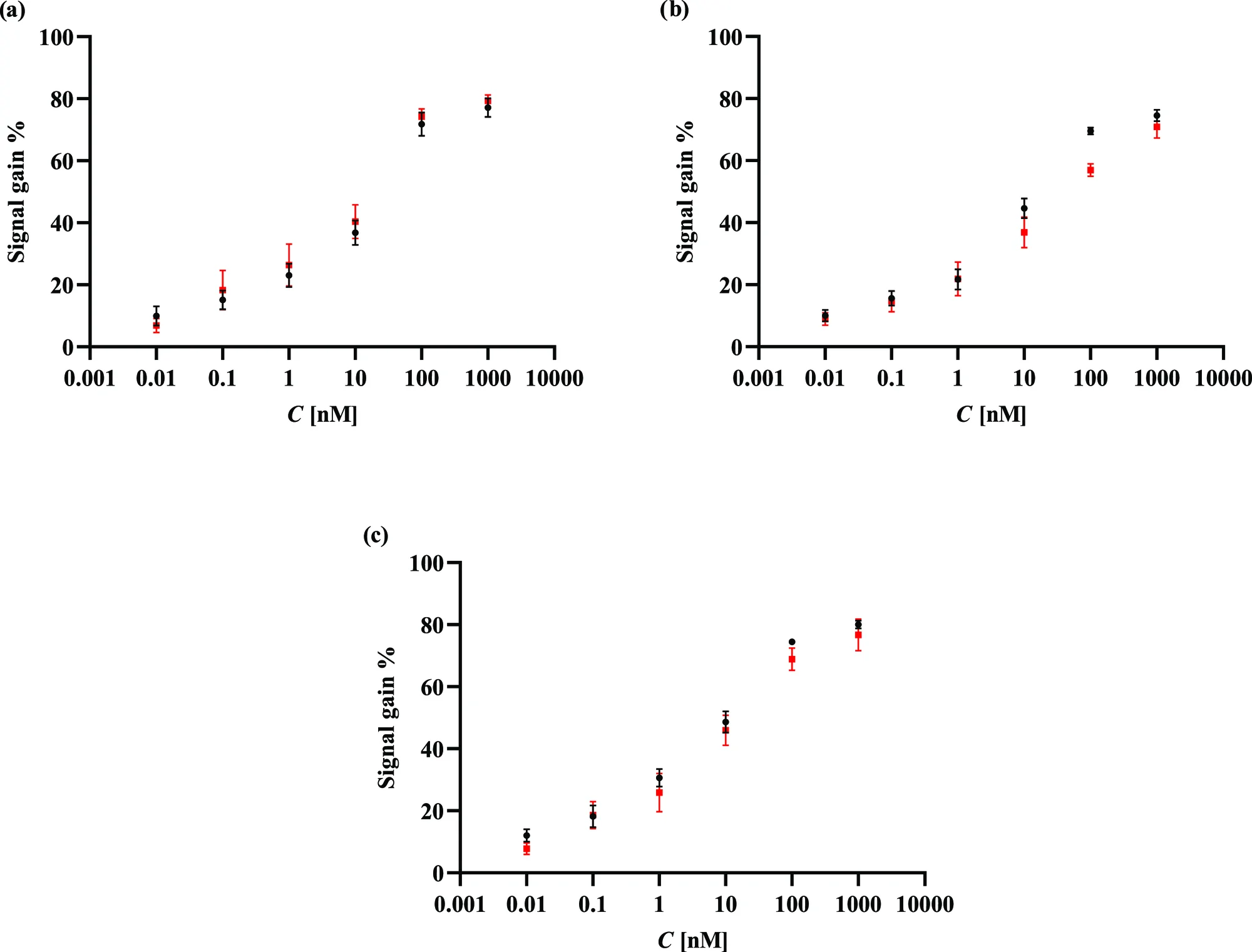
Titration curves obtained using E-DNA sensors to detect DNA and miRNA targets corresponding to (a) miRNA-146b, (b) miRNA-221 and (c) miRNA-223, prepared in PBS buffer. The biosensor response is represented as signal gain against increasing target concentrations (0.01 nM – 1000 nM). Values represent the average signal gain obtained from three independent sensors, with error bars indicating the standard deviation.

The resulting titration curves were fitted to the Langmuir-Hill equation^60^ using KaleidaGraph software (Figures S2–S4). The *R* ^2^ values of the fittings, along with the obtained equilibrium dissociation constants (*K*_d_) are listed in Table 2.

**Table 2:** Dissociation Constants (*K*_d_) and Correlation Coefficients (*R* ^2^) of the Fitting of the Titration Curves for DNA and miRNA Targets Depicted in Figure 3 to the Langmuir-Hill Equation^a^

	DNA target		miRNA target	
biomarkers	*K*_d_ (nM)	R^2^	*K*_d_ (nM)	*R* ^2^
miRNA-146b	3.0 ± 0.8	0.988	2.5 ± 0.4	0.993
miRNA-221	2.0 ± 0.4	0.990	2.2 ± 0.4	0.992
miRNA-223	2.3 ± 0.4	0.992	3.5 ± 0.6	0.997

a The extracted *K*_d_ values from the resulting fits are within experimental error of one another, indicating no statistically meaningful differences in binding affinity between the targets.

### 3.4 Milk Analysis Using E-DNA Sensors to Diagnose Bovine Mastitis

To demonstrate the diagnostic potential of the developed panel of E-DNA sensors, we evaluated whether the individual electrochemical responses of three miRNA-specific sensors could reliably distinguish milk samples from healthy cows and those with subclinical mastitis. Rather than relying on a single biomarker, which may be influenced by individual variability or unrelated physiological conditions,^61,62^ we focused on detecting the specific miRNA signature, comprising miRNA-146b, miRNA-221 and miRNA-223, previously identified through NGS as associated with the early, subclinical stage of mastitis. This strategy enables a more nuanced molecular diagnosis by capturing the disease-specific expression profile.^63,64^

Detecting miRNAs in milk presents considerable challenges due to the biological complexity of the matrix, which contains high concentrations of proteins, lipids, lactose, minerals, and endogenous enzymes. These components can interact with nucleic acids or nonspecifically adsorb onto the sensor surface, clog active sites, and interfere with electron transfer processes, thereby reducing the analytical performance of the sensor.^65,66^ Furthermore, miRNAs are often bound to proteins or encapsulated within microvesicles, which serve to protect them from RNase degradation.^67^ Effective detection thus requires liberation of miRNAs from these protective structures while simultaneously safeguarding them from RNase activity. To address these issues, we used a straightforward extraction protocol to isolate RNA from milk prior to analysis. This sensing approach is simple, rapid, and does not require amplification steps such as PCR, making it suitable for prompt diagnostics. We focused on detecting the identified miRNA signature using this integrated strategy, which effectively overcomes the challenges posed by the complex milk matrix.

RNA samples extracted from the milk of five healthy cows and five cows identified as suffering subclinical mastitis, were electrochemically analyzed using the developed sensors. Prior to analysis, the samples were diluted 1:1 in PBS. The average signal gain for miRNA-146b was 6% in healthy samples and 20% in subclinical samples, with the difference being statistically significant (*p* < 0.05, unpaired *t* test). Similarly, for miRNA-221, the average signal gain was 7% in healthy samples and 19% in subclinical samples, also showing a significant difference. For miRNA-223, healthy and subclinical samples exhibited average signal gain of 10 and 16%, respectively. This difference did not reach statistical significance, which may reflect the limited statistical power associated with the small sample size attained in a study comprising naturally infected cows (Figure 4). It should be noted that while miRNA-223 showed the largest increase in expression based on sequencing data, the corresponding sensor exhibited a comparatively lower gain. This discrepancy may be explained by biological variability rather than limitations of sensor performance. MiRNA expression levels are highly dynamic and can fluctuate significantly between sampling rounds due to variations in animal physiological status, disease progression, inflammatory stage, environmental factors, and sample collection timing.^68^ As previously described, milk samples used for NGS analysis and sensing tests were collected from different animals and during different seasons, what supports the variations observed in miRNA expression and sensing results. Importantly, these findings demonstrate that the combined response of the three sensors can reliably differentiate healthy from subclinical cows by detecting variations in miRNA expression levels. This result underscores the platform’s potential for the prompt diagnosis of subclinical mastitis. The repeatability of the platform was evaluated by calculating the relative standard deviation (RSD%), which was found to be less than 3% (*n* = 5) for all sensors.

**Figure 4:**
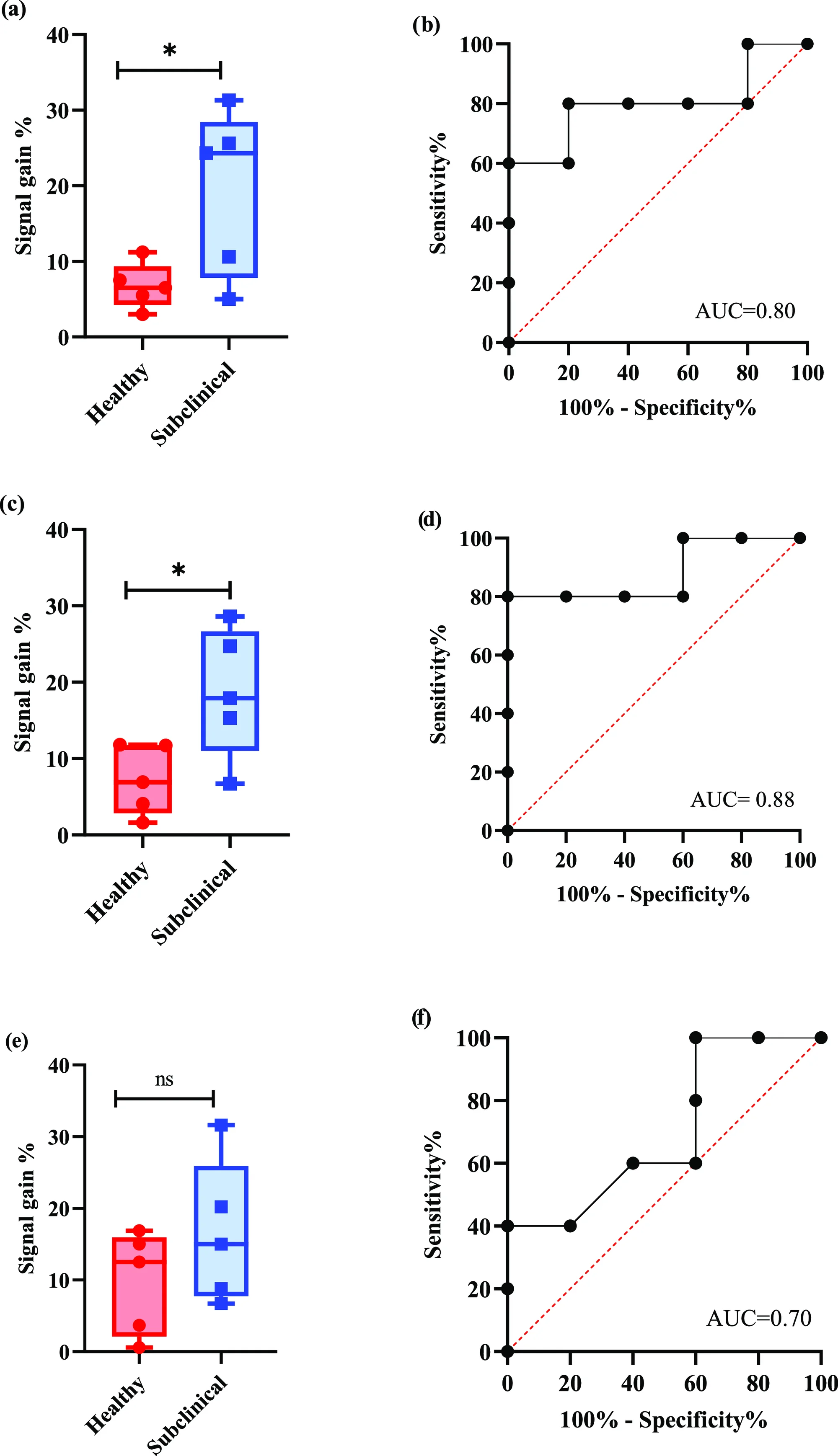
Signal gain and corresponding ROC curves for milk-derived RNA extracts (diluted 1:1 in PBS) obtained from five healthy cows and five cows with subclinical mastitis, using E-DNA sensors targeting miRNA-146b, miRNA-221, and miRNA-223. Panels (a), (c) and (e) show the signal gain obtained for miRNA-146b, miRNA-221, and miRNA-223, respectively, where values represent the average signal gain obtained from five independent sensors for each test group, with error bars indicating the standard deviation. Statistical significance was determined using an unpaired *t* test, with *p* ≥ 0.01 (*) showing significant difference between groups and ns denoting no significant difference. Panels (b), (d) and (f) present the corresponding ROC curves for miRNA-146b, miRNA-221, and miRNA-223, respectively. The AUC values in the ROC curves were determined by using Wilson/Brown test with 95% confidence interval.

In addition, Receiver Operator Characteristic (ROC) curves were plotted to further assess the ability of the developed sensor platform to discriminate between subclinical and healthy samples based on miRNA detection. ROC analysis is a widely used method for evaluating diagnostic test performance. The ROC curve plots sensitivity (true positive rate) against (1 – specificity) (false positive rate), with the area under the curve (AUC) providing a quantitative measure of diagnostic performance. AUC values range from 0.5 (no discrimination) to 1 (perfect discrimination).^60^ The optimal cutoff point was determined using Youden’s Index (sensitivity + specificity – 1), which maximizes the combined sensitivity and specificity, and this threshold was incorporated into the logistic regression model. As shown in Figure 4, the sensors targeting miRNA-221 and miRNA-146b achieved AUC values of 0.88 and 0.80, respectively, demonstrating strong discriminatory performance. The sensor for miRNA-223 yielded an AUC of 0.70, indicating moderate performance. These results further support that the developed sensors can reliably detect diagnostically relevant miRNAs in milk and differentiate subclinical mastitis cases from healthy ones.

In summary, we have successfully demonstrated that the panel of three developed E-DNA sensors can accurately distinguish between healthy and subclinical bovine mastitis cases by detecting a specific miRNA expression signature. Results support the primary objective of our study, i.e., to develop a sensitive, reliable, and amplification-free method for early mastitis detection. These findings underscore the promise of miRNA-targeted electrochemical sensors as a diagnostic approach for the early identification and monitoring of subclinical mastitis in dairy herds.

### 3.5 Correlation of SCC, Bacterial Load and miRNA Responses

To determine the diagnostic relevance of the electrochemical sensor responses, we analyzed whether the signal gain for the three miRNA targets exhibited consistent trends with established biological indicators of mastitis, i.e., SCC and TBC. The objective was not to directly correlate sensor outputs with these conventional markers but to evaluate whether the molecular signals detected by the sensor reflect the biological patterns associated with infection (bacterial growth) and inflammation (increased SCC) that characterize mastitis progression.

Correlation analysis using Spearman’s rank correlation (ρ) was performed to evaluate whether these molecular markers not only distinguish healthy from subclinical animals but also track the underlying inflammatory and microbial dynamics of mastitis (Figure 5). A strong positive correlation was observed between SCC and TBC (ρ = 0.74, *p* = 0.019), consistent with SCC’s role as an indicator of intramammary infection. This association arises because invading bacteria trigger the recruitment of neutrophils into the mammary gland, which elevates SCC.^69–71^ However, it is important to recognize that SCC lacks disease specificity, as increases may also occur due to noninfectious factors such as lactation stage, stress or udder trauma.^72,73^

**Figure 5:**
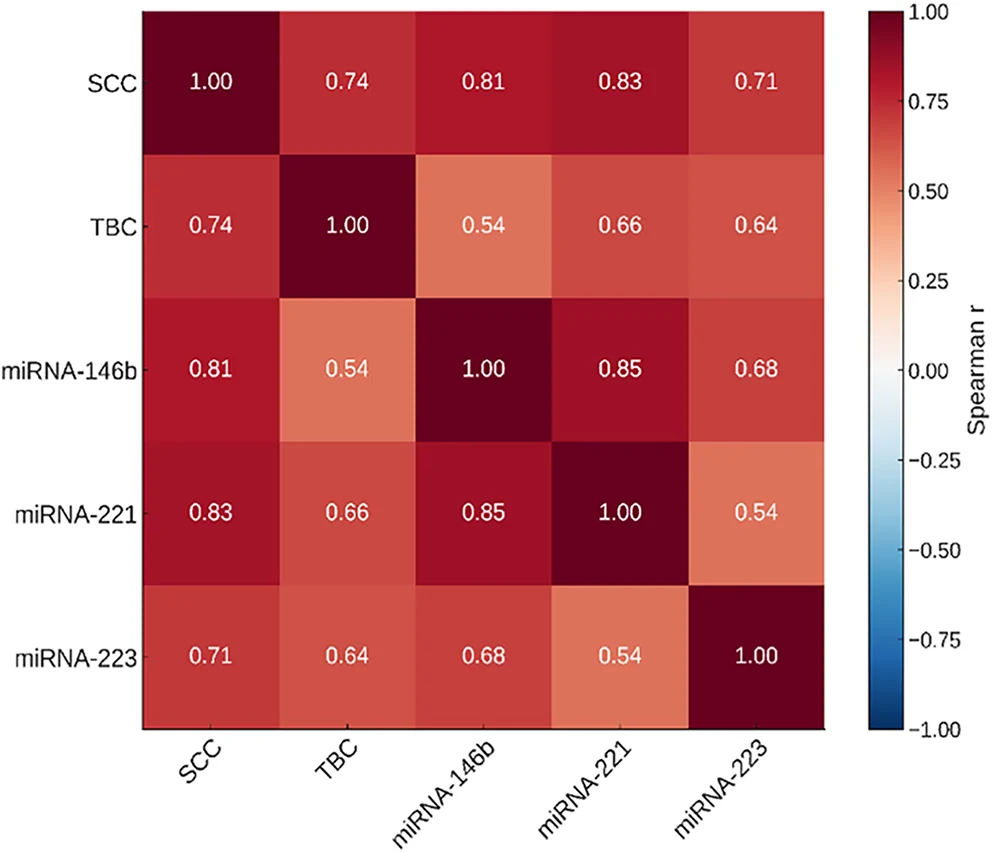
Heatmap of Spearman’s correlation coefficients between SCC (cells/mL), total bacterial count (TBC, log_10_ CFU/mL), and electrochemical sensor responses to milk-derived miRNAs (miRNA-146b, miRNA-221 and miRNA-223). Positive correlations are represented by darker red shading, with values approaching 1.0 indicating stronger associations.

Importantly, SCC showed strong correlation with miRNA-221 (ρ = 0.83, *p* = 0.015) and miRNA-146b (ρ = 0.81, *p* = 0.022), while miRNA-223 showed a positive but only borderline association (ρ = 0.71, *p* = 0.058). In mastitis, exosomal miRNA-221 released from mammary epithelial cells activates macrophages via SOCS1/STAT1/STAT3 signaling, which drives cytokine production thus amplifying inflammation.^74,75^ Activated macrophages release TNF-α, IL-1β and IL-6, which in turn drive immune cell recruitment.^76^ In other diseases, miRNA-221 has also been linked to immune-cell migration.^77^ In cows, miRNA-221 is upregulated in mastitic milk and positively associated with SCC,^50,78^ consistent with our findings.

Previous studies demonstrated that miRNA-146b is significantly upregulated in mastitis-affected mammary tissues, where it targets TRAF6 and IRAK1, key regulators of TLR/NF-κB signaling.^79,80^ Although miRNA-146b is typically described as a negative feedback regulator of inflammation, its expression rises alongside SCC during active mastitis, consistent with our results.^80^ This suggests that miRNA-146b is coinduced with inflammation, while its inhibitory role likely becomes more apparent during resolution. Importantly, these observations highlight a critical gap: the temporal dynamics of miRNA-146b in naturally occurring mastitis remain poorly understood. Further longitudinal studies are needed to clarify whether its role shifts from pro-inflammatory association in early infection to negative regulation during recovery.

For miRNA-223, experimental mastitis models have highlighted its protective role, where its overexpression acts as a predominant anti-inflammatory regulator, reducing disease progression by targeting CBLB to inhibit the PI3K/AKT/NF-κB pathway and by downregulating the NLRP3 inflammasome and Keap1/Nrf2 signaling.^48,81^ In contrast, studies on naturally occurring mastitis have emphasized its value as a biomarker of inflammation. For example, Tzelos et al.^22^ showed that miRNA-223 in whole milk correlated with inflammatory mRNAs and rose stepwise with California Mastitis Test (CMT) score, achieving high accuracy in distinguishing healthy from mildly inflamed quarters.

The weaker correlations in our study may reflect the greater heterogeneity of naturally occurring mastitis cases, including variation in pathogen exposure and stage of infection, compared with the more consistent responses seen in induced models. It is plausible that, similar to miRNA-146b, miRNA-223 expression rises alongside SCC early in infection (diagnostic phase) but later exerts anti-inflammatory feedback to dampen excessive immune responses (regulatory phase). However, no studies to date have comprehensively explored this temporal regulation in natural mastitis cases. Addressing this knowledge gap will be essential to dissect miRNA-223’s true diagnostic and prognostic value.

Additionally, these three miRNAs demonstrated interrelationships. miRNA-146b showed strong positive correlations with both miRNA-221 (ρ = 0.85, *p* = 0.003) and miRNA-223 (ρ = 0.68, *p* = 0.035), suggesting coordinated regulation during mastitis, likely through shared inflammatory signaling pathways. In contrast, miRNA-221 and miRNA-223 were only weakly correlated (ρ = 0.54, *p* = 0.11), consistent with their more distinct roles described above. These patterns indicate that miRNA-146b may function as a central node of regulation, whereas miRNA-221 and miRNA-223 appear to be more independently regulated.

Overall, these findings support the diagnostic and to some extent prognostic potential of the selected miRNA panel. While SCC and bacterial counts remain valuable indicators, miRNA biomarkers can provide an additional molecular layer that directly reflects host–pathogen interactions. In particular, miRNA-221 and miRNA-146b show strong promise as adjunct indicators for early mastitis detection and for monitoring disease severity in subclinical cases.

When TBC values were compared with sensor responses, miRNA-221 was significantly associated with CFU levels, while miRNA-223 showed a marginal correlation and miRNA-146b exhibited only a weak, nonsignificant trend. This pattern suggests that miRNA-221, and to some extent miRNA-223, may serve as indicators of microbial burden. Consistent with this trend, exosomal miRNA-221 from bovine mammary epithelial cells has been reported to be strongly induced by *S. aureus* ligands and promote macrophage activation,^75^ while other experimental studies showed that higher intramammary *S. aureus* CFU loads are associated with greater induction of miRNA-223.^48^ In contrast, in our study miRNA-146b showed no relationship with bacterial counts, and we found no evidence in the literature describing such an association. Instead, its consistent upregulation in mastitis-affected mammary tissue, as observed both in our data and in previous studies,^79,80^ supports the interpretation that miRNA-146b primarily reflects host inflammatory signaling rather than microbial burden.

## 4 Conclusions

We first collected milk samples from healthy cows and cows naturally infected with subclinical mastitis. Using NGS we then identified a distinct miRNA expression profile, with overexpressed miRNA-146b, miRNA-221 and miRNA-223, in the milk of infected cows. The levels of the three identified miRNAs in milk could be used to effectively discriminate healthy cows from cows with subclinical mastitis, establishing a novel molecular signature for early mastitis detection. Next, we engineered and optimized a panel of E-DNA sensors to detect these miRNAs directly from milk-derived RNA extracts without amplification or extensive sample preparation. The sensors demonstrated high signal gains, reproducible responses, and selective target recognition, even in the presence of noncomplementary and 2-base pair mismatch sequences, confirming their suitability for the analysis of complex biological matrices. ROC analysis further supported the diagnostic performance of the sensors, with high AUC values indicating strong discriminatory power. To confirm biological relevance, sensor responses were evaluated against established mastitis indicators. miRNA-221 and miRNA-146b showed statistically significant positive associations with SCC, reflecting their link to host inflammatory activity, while miRNA-221 and miRNA-223 were further associated with TBC, suggesting their relationship with microbial burden. These findings demonstrate that the sensors not only differentiate healthy from subclinical mastitic cows but also, to some extent, track the inflammatory and microbial dynamics underlying infection. Importantly, this host-response-based strategy overcomes limitations of conventional diagnostics, where bacterial detection or elevated SCC can occur in the absence of active disease.

Overall, our findings highlight the strong diagnostic potential of E-DNA sensors targeting a panel of host immune-derived biomarkers previously identified in the milk of naturally infected cows, as noninvasive, rapid, low-cost, and field-deployable tools for early mastitis diagnosis. Future work will focus on fully integrating sample preparation and detection into a streamlined, automated sample-to-answer platform, enabling on-site use, real-time decision-making, and timely intervention to improve health management globally.

## Supplementary Material



## Data Availability

NGS data, including UMI-based sequencing quality control reports and miRNA expression data, are available at OSF via our view-only project link: https://osf.io/3pnr5/overview?view_only=3e23e25267f54b2281c233c6eb5866bb

## Notes

Detailed procedures for the collection, handling, and classification of milk samples, the optimization of hybridization conditions, the nonlinear regression analyses of titration curves fitted using the Langmuir–Hill isotherm, and the full sequences of all oligonucleotides used in sensor design (PDF)
