# Supplementary material for: Amplification-Free Detection of MiRNA Signatures in Milk: A Noninvasive Sensing Approach for Early Diagnosis of Bovine Mastitis
Source: ACS Meas Sci Au. 2026 Jun 17;6(4):1091–104. doi: 10.1021/acsmeasuresciau.6c00060 (PMC13495468; doi:10.1021/acsmeasuresciau.6c00060)
Supplement: Supplementary file 1 [file tg6c00060_si_001.pdf]

Supporting Information

Amplification-Free Detection of MiRNA Signatures in Milk: A Non-Invasive Sensing Approach for Early Diagnosis of Bovine Mastitis

*Kandeel Shafique,[1,2] Hedieh Haji-Hashemi,[1] Łukasz Gontar,[3] Māra Pilmane,[4] Gergely Maróti,[5,6] Beatriz Prieto-Simón[1,7]**

[1] Institute of Chemical Research of Catalonia, The Barcelona Institute of Science and Technology, Av. Països Catalans 16, 43007 Tarragona, Spain

[2] Department of Electronic Engineering, Universitat Rovira i Virgili, 43007 Tarragona, Spain

[3] Research and Innovation Centre Pro-Akademia, 95-050 Konstantynów Łódzki, Poland

[4] Institute of Anatomy and Anthropology, Rîga Stradiòð University, 9 Kronvalda Blvd., LV-1010 Rîga, Latvia

[5] SeqOmics Biotechnology Ltd., 6782 Morahalom, Hungary

[6] Institute of Plant Biology, HUN-REN Biological Research Center, 6726 Szeged, Hungary

[7] ICREA, Pg. Lluís Companys 23, 08010 Barcelona, Spain

Corresponding author: bprieto@iciq.es

Table of contents

1. Classification of milk samples according to SCC levels 2
2. Optimization of hybridization buffer 10
3. Titration curves fitting 11
4. Oligonucleotide sequences 13

1. Classification of milk samples according to SCC levels

Cows were classified according to their health status based on the following criteria: healthy – SCC < 200000 cells/mL with no clinical signs, and subclinical – SCC 200000–500000 cells/mL with no clinical signs.

Table S1. SCC values and sample assignment for the first round of milk collection. Total RNA extracted from these samples was pooled and used for NGS-based profiling.

Cow ID	Quarter	Group	Day 1	Day 2	Day 3
1501	A		238890	213838	210124
1501	B		2552543	1567088	636062
1501	C		273088	348631	393343
1501	D		355967	4330453	2598338
1512	B		2993737	1208966	1573005
1512	C		107685	25996	6709
1512	D		14631	10320	18546
1515	A		17636	14637	10630
1515	C		15907	19993	23641
1515	D		318096	159052	147928
1516	A	Subclinical	144347	325600	275315
1516	B		15205	36961	9954
1516	C		11578	10519	10028
1516	D		22390	53850	12871
1524	A	Subclinical	52491	26280	10146
1524	B		16213	9205	11526
1524	C		1084948	762782	499575
1524	D		13733	3011	11114
1531	A		3386	5228	2674
1531	B		3142	2560	2755
1531	C		3706	5648	9659
1531	D		9823	11518	1693
2750	A		212992	141424	218233
2750	B		161053	118588	154371
2750	C		137397	87454	146104
2750	D	Subclinical	252897	126138	289091
2754	A		19404	14137	12313
2754	B		26093	15892	9056

2754	C		44139	41801	22439
2754	D		25411	20169	14543
2759	A	Healthy	4908	3933	7308
2759	B		11722	11534	8286
2759	C		7418	5823	4732
2759	D		10363	12673	8048
2760	A		1825590	1143633	582200
2760	B		14357	19640	5130
2760	C		148539	78127	87899
2760	D		5152359	903688	6442159
2762	A		649245	98970	107139
2762	B		85136	19215	38553
2762	C		16103	15957	7010
2762	D		2284	13174	7953
2764	A		59472	2586	6909
2764	B		81996	29123	43009
2764	C		18205	2908	12052
2764	D		14241	5873	6866
2765	A	Subclinical	13715	13106	16948
2765	B		17673	35272	18270
2765	C		377674	228709	611057
2765	D		52519	45345	123473
2779	A		22019	28679	20696
2779	B		28981	13885	15170
2779	C		5125	13922	20329
2779	D		7580	7594	15864
2789	A		9625	7890	14138
2789	B		47873	26119	40834
2789	C		4502	687	7331
2789	D		3483	3219	11187
2790	A		9844	5269	5196
2790	B		33519	14160	26758
2790	C		8106	6894	9834
2790	D		8014	12140	11632
2792	A		8430	6956	3626
2792	B		11522	5941	5893
2792	C		8464	7937	3347

2792	D		17875	8600	5528
2794	A		7283	8425	10115
2794	B		176005	126113	130950
2794	C		1200053	502811	3115277
2794	D		100748	46338	82041
2796	A		43573	54675	57910
2796	B		12556	6264	12130
2796	C		156235	228920	157634
2796	D		8204	72667	33979
4302	A		1915	3145	4987
4302	B		4102	3147	5528
4302	C		6286	2211	3865
4302	D		5514	5191	2471
4304	A		17212	11237	9481
4304	B		16463	16198	42686
4304	C		4326046	6570846	2274798
4304	D		14815	11139	28314
4305	A		4661	2128	4698
4305	B		3030	3765	3526
4305	C		11235	6849	3928
4305	D		6920	10491	2885
4308	A		1573	1092	1681
4308	B		2245	3096	4283
4308	C		2848	2274	3847
4308	D		2433	25479	11591
4310	A		4236	5396	6797
4310	B	Healthy	72181	32142	58277
4310	C		12684	4426	8203
4310	D		8135	8056	12991
4311	A		45776	24692	50151
4311	B		55581	12252	36104
4311	C		52237	80431	331640
4311	D		22408	20041	189518
4313	A		6250	5675	9986
4313	B		11957	11850	5429
4313	C		36328	42370	11024
4313	D		21747	23039	14530

4315	A	Healthy	5680	2673	6462
4315	B		15155	11404	3349
4315	C		1105	21788	4285
4315	D		24226	10308	9780
4316	A		11415	1122	2473
4316	B		10529	14031	6152
4316	C		17442	12388	5135
4316	D		29603	16795	13531
4317	A		15692	11103	2564
4317	B		25679	16392	3693
4317	C		401566	261692	123134
4317	D		8545	17371	2319
4318	A	Healthy	1641	1825	1613
4318	B		8969	6538	7892
4318	C		2498	4535	4521
4318	D		8038	5151	10382
4319	A		13840	41160	46041
4319	B		4144	10043	17051
4319	C		8456	15025	21595
4319	D		25943	19864	28706
4320	A		14111	9077	16746
4320	B		11812	14522	10245
4320	C		4107	9108	4003
4320	D		114391	50555	71742
4322	A		55680	21110	23562
4322	B		77432	30331	41330
4322	C		104864	54412	80813
4322	D		72269	24822	82693
4323	A	Healthy	11054	30762	18470
4323	B		30313	10471	16796
4323	C		10512	20018	14192
4323	D		26609	63333	22602
4324	A		97251	47784	57803
4324	B		142902	130277	143432
4324	C		25800	28897	65729
4324	D		28555	10586	31884
4325	A		48898	34143	38843

4325	B		57386	55061	40967
4325	C		86934	41085	56674
4325	D		76714	92088	86054
4327	A	Subclinical	22448	28656	5739
4327	B		114295	168413	34767
4327	C		36834	498251	38992
4327	D		520692	496088	655012
4328	A		12138	18235	37532
4328	B		55060	9778	16530
4328	C		36652	24392	27771
4328	D		248589	235445	143966

Table S2. SCC values and final sample assignment for the second round of milk collection. Total RNA extracted from these samples was used to assess the diagnostic accuracy of the E-DNA sensing results.

Cow ID	Quarter	Group	Day 1	Day 2	Day 3	Day 4	Day 5	Day 6
2761	A		568	1051	2100			
2761	B		5883	12587	3072			
2761	C		186899	101114	337325			
2761	D		287192	765487	306415	328440	143162	
2764	A		106885	45911	108912			
2764	B	Subclinical	2740457	662164	441170	295612	326118	96704
2764	C		44312	39528	30532			
2764	D		62489	20414	79074			
2790	A		4116	2511	2053			
2790	B		4874	2709	5609			
2790	C	Healthy	60836	75086	15577	8111		
2790	D		7989	3092	0			
4303	A		18161	3928	3089			
4303	B		9533	3262	5177			
4303	C		630379	686750	407068	69704		
4303	D		78946	34675	53050			
4304	A		7707	4693	13521			
4304	B		4531	1576	4114			

4304	C	Subclinical	1923267	902791	505028	1246291	439856	523448
4304	D		351533	411756	231481			
4305	A		13041	64936	74598			
4305	B		7587	1521	10671			
4305	C		33573	17897	106692			
4305	D		248215	2119976	628037	132952		
4308	A		7076	2769	1907			
4308	B		13522	6304	4074			
4308	C	Subclinical	843871	339587	293576	100866		270555
4308	D		9652	9605	4009			
4313	A		221904	152527	228356			
4313	B		129191	69344	49477			
4313	C		77526	27368	9502			
4313	D	Subclinical	1610369	743524	4482709	2768207	95291	1134
4314	A		170335	68099	34860			
4314	B		204093	145495	80578			
4314	C		35487	44871	41447			
4314	D		4620	1833	5118			
4320	A		38769	33957	34967			
4320	B		37087	35379	25911			
4320	C		72628	79621	50541			
4320	D		246316	356177	215719			
4321	A		33564	23367	23587			
4321	B		170825	84400	63461			
4321	C		22597	31427	19691			
4321	D		45895	28934	19118			
4323	A		96370	182574	160434			
4323	B		143355	220466	153967			
4323	C		50044	97901	49692			
4323	D		148993	204111	175230			
4325	A		18332	7791	15858			
4325	B		49332	15953	53600			
4325	C		9483	14270	21244			
4325	D		85793	135327	94681			
4328	A		31419	22127	32718			
4328	B		286007	222591	230540			
4328	C		88122	52788	90963			

4328	D		9297	9693	18178			
4329	A		11355	9655	8783			
4329	B		9857	2196	3872			
4329	C		36805	20123	15649			
4329	D		13820	6822	12492			
4332	A		11257	34360	27854			
4332	B		5960	24145	35315			
4332	C		93260	112672	109014			
4332	D		54822	63341	76226			
4334	A		4054	21017	8159			
4334	B		904292	1084066	607244			
4334	C		5538	9520	13173			
4334	D		1932492	994249	715938	962338	333217	57275
4335	A		3536	3116	0			
4335	B		8120	9438	14540			
4335	C		4810	0	3888			
4335	D		8489	14126	13481			
4336	A		18038	15992	30610			
4336	B		9965	7866	27875			
4336	C		13769	3056	17807			
4336	D		10072	12591	5453			
4337	A		118079	65577	117597			
4337	B		131553	91134	254173			
4337	C		441693	334439	344208			
4337	D		231823	195650	353740			
4338	A		2001	4152	0			
4338	B		2294	3611	6457			
4338	C		7964	5042	2289			
4338	D	Healthy	9029	3970	8536	1071		
7710	A		2344	2352	516			
7710	B		2095	0	2023			
7710	C		7301	3180	3129			
7710	D	Healthy	7374	6921	1677	0		
7714	A		5177	2781	2244			
7714	B		12504	2729	9232			
7714	C		26863	2547	4106			
7714	D		23357	11955	10747			

7716	A		5049	2880	0			
7716	B		5762	3026	0			
7716	C	Healthy	3601	4488	7806	0		
7716	D		10614	8457	7544			
7718	A		8112	8566	5552			
7718	B		20648	4925	7619			
7718	C		16971	19800	3654			
7718	D		188162	147740	147875			
7721	A		79212	6253	4951			
7721	B		128429	3866	5506			
7721	C		16651	6271	7396			
7721	D		11830	11505	10751			
7722	A		10161	6294	6246			
7722	B	Healthy	10525	5595	9204	3422		
7722	C		5538	12080	4191			
7722	D		8054	5440	0			
7724	A		124620	27627	52984			
7724	B		167157	65783	13736			
7724	C		132382	92711	11667			
7724	D		141120	39119	10749			
7725	A		65000	29678	28651			
7725	B		128729	69519	63267			
7725	C		16828	14508	23682			
7725	D		61448	14169	46165			
7726	A		26489	8199	3626			
7726	B		19249	18401	10365			
7726	C		9173	8372	5999			
7726	D	Subclinical	1862604	2254179	2682946	1474425	1702492	
7727	A		317353	402641	122607			
7727	B		2183	9346	7699			
7727	C		227050	357391	2121500			
7727	D	Subclinical	595828	401293	2433893	672620	131247	158453
7728	A		131133	60938	61548			
7728	B		56383	29167	16945			
7728	C		73383	15660	17426			
7728	D		45721	43956	17859			
7730	A		12449	4538	6654			

7730	B		10715	7658	7677		
7730	C		17002	5737	5103		
7730	D		640314	313398	600622	305219	138462
7735	A		493338	466902	375009		
7735	B		4736	4821	11583		
7735	C		17154	15097	8302		
7735	D		10595	12167	8575		

Table S3. SCC values and total bacterial count of selected milk samples from second collection round.

Cow ID	Quarter	Group	SCC level [cells/ml]	Total bacterial count (log_{10} CFU/mL)
2790	C	Healthy	8111	2.43
4338	D	Healthy	1071	2.73
7710	D	Healthy	0	1.82
7716	C	Healthy	0	2.78
7722	B	Healthy	3422	2.30
4313	D	Subclinical	95291	3.34
7726	D	Subclinical	1702492	3.30
2764	B	Subclinical	96704	3.37
4304	C	Subclinical	523448	3.55
4308	C	Subclinical	270555	2.87

2. Optimization of hybridization buffer

To determine the most suitable buffer for nucleic acid hybridization, we compared two commonly used buffers, PBS and Tris, using the miRNA-223 sensor. Both buffers support hybridization, but differences in ionic strength can influence hybridization efficiency and specificity. Sensors functionalized with a capture probe complementary to miRNA-223, alongside control sensors with non-specific probes, were incubated with a 100 nM solution of the DNA analog of miRNA-223. The electrochemical response before and after hybridization were measured to assess performance. Both buffers demonstrated good hybridization, with PBS showing slightly higher signal gain (~59%) and lower non-specific response (~4%) compared to Tris (~50% hybridization efficiency

and ~13% non-specific response). Although these differences are not statistically meaningful and fall within experimental uncertainty, PBS was selected to perform all subsequent experiments.

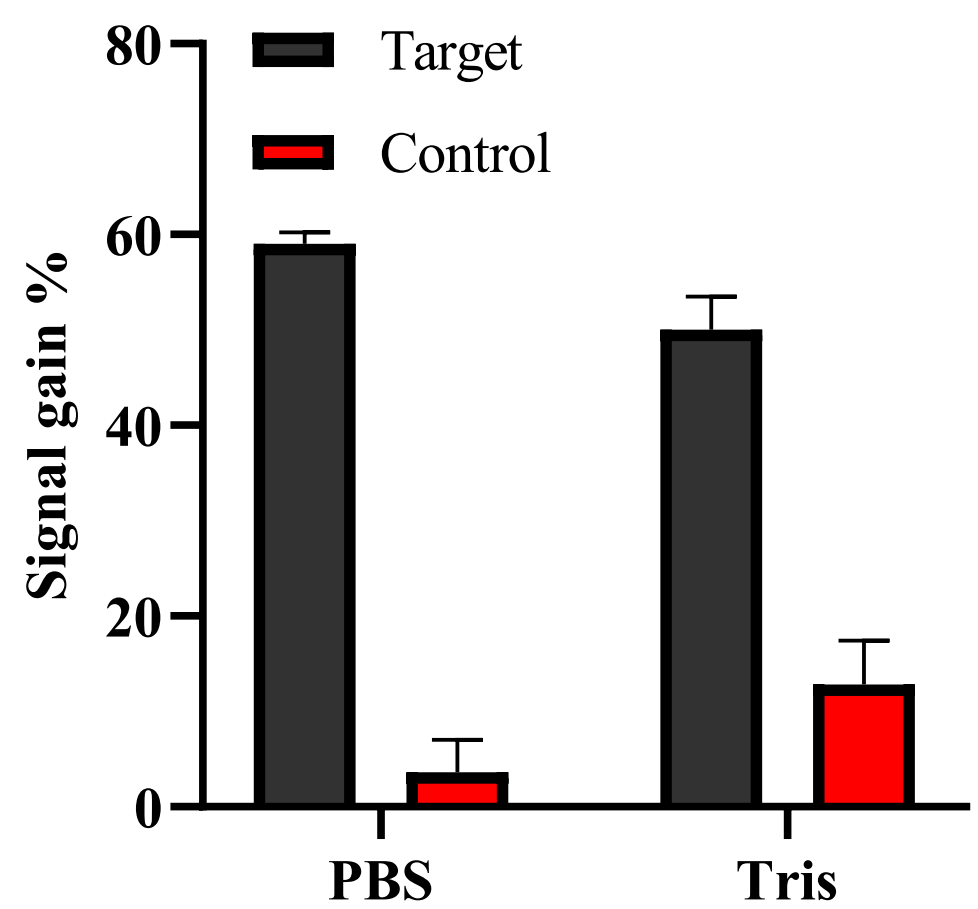

Figure S1. Effect of buffer on DNA hybridization illustrated as the signal gain measured by E-DNA sensors. Sensors were modified with a capture probe for miRNA-223 and controls with a non-specific capture probe, respectively. The response was recorded for a 100 nM DNA target, with a sequence complementary to the miRNA-223 capture probe, prepared in PBS and Tris buffers. Values represent the average signal gain obtained from three independent sensors for each test group, with error bars indicating the standard deviation.

3. Titration curves fitting

To determine apparent sensor dissociation constants, we performed non-linear regression analyses against the Langmuir-Hill isotherm.

$$\text{Signal gain \%} = \gamma \frac{[T]^n}{Kd + [T]^n} \qquad \textbf{(1)}$$

where n is the number of aptamer binding sites (1 for all cases considered here), γ is the maximum signal output, and Kd is the apparent dissociation constant. KaleidaGraph software was used for curve fitting.[1] Curve fittings are shown in **Figures S2-S4.**

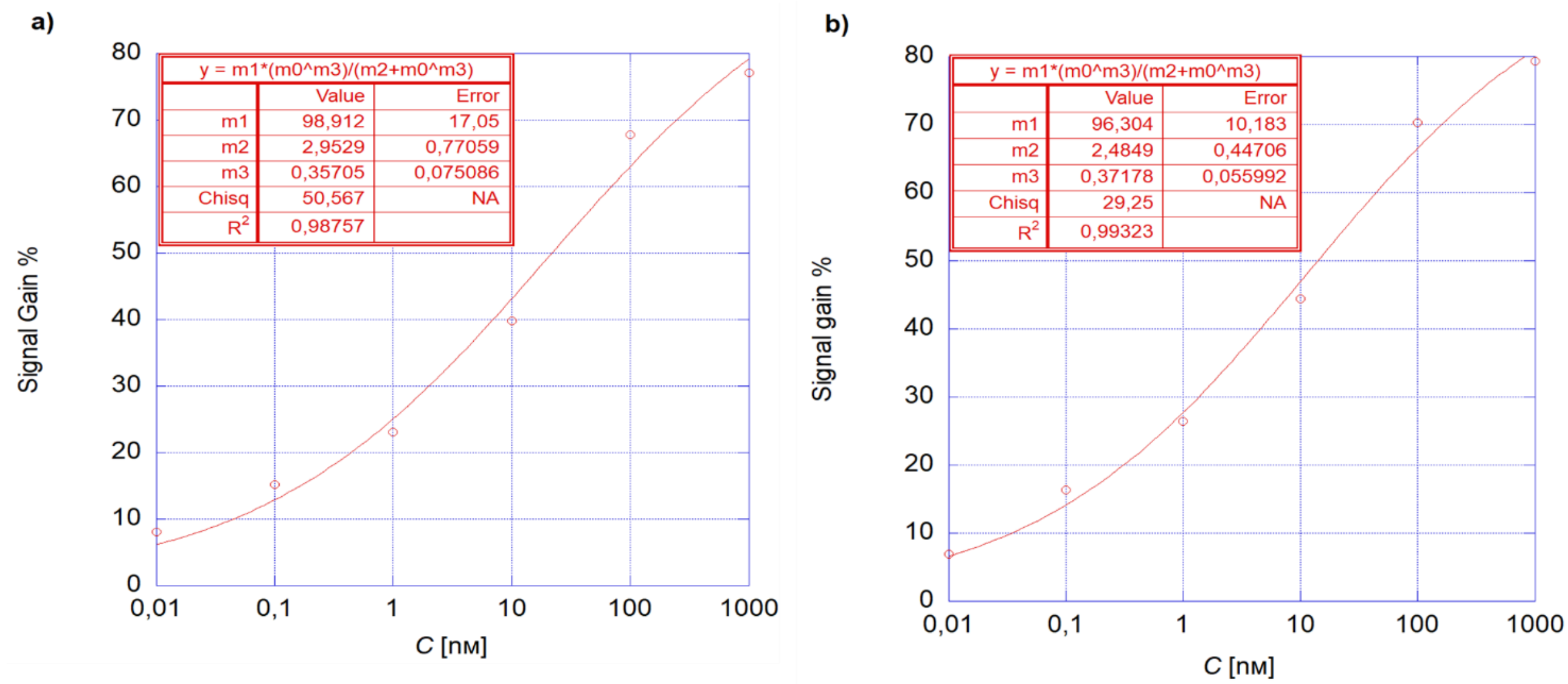

Figure S2. Langmuir-Hill isotherm fit of the miRNA-146b sensor response to(a) DNA and (b) miRNA targets in buffer. Inset table show the estimated maximum signal gain (m_1) and dissociation constant (Kd, denoted as m_2).

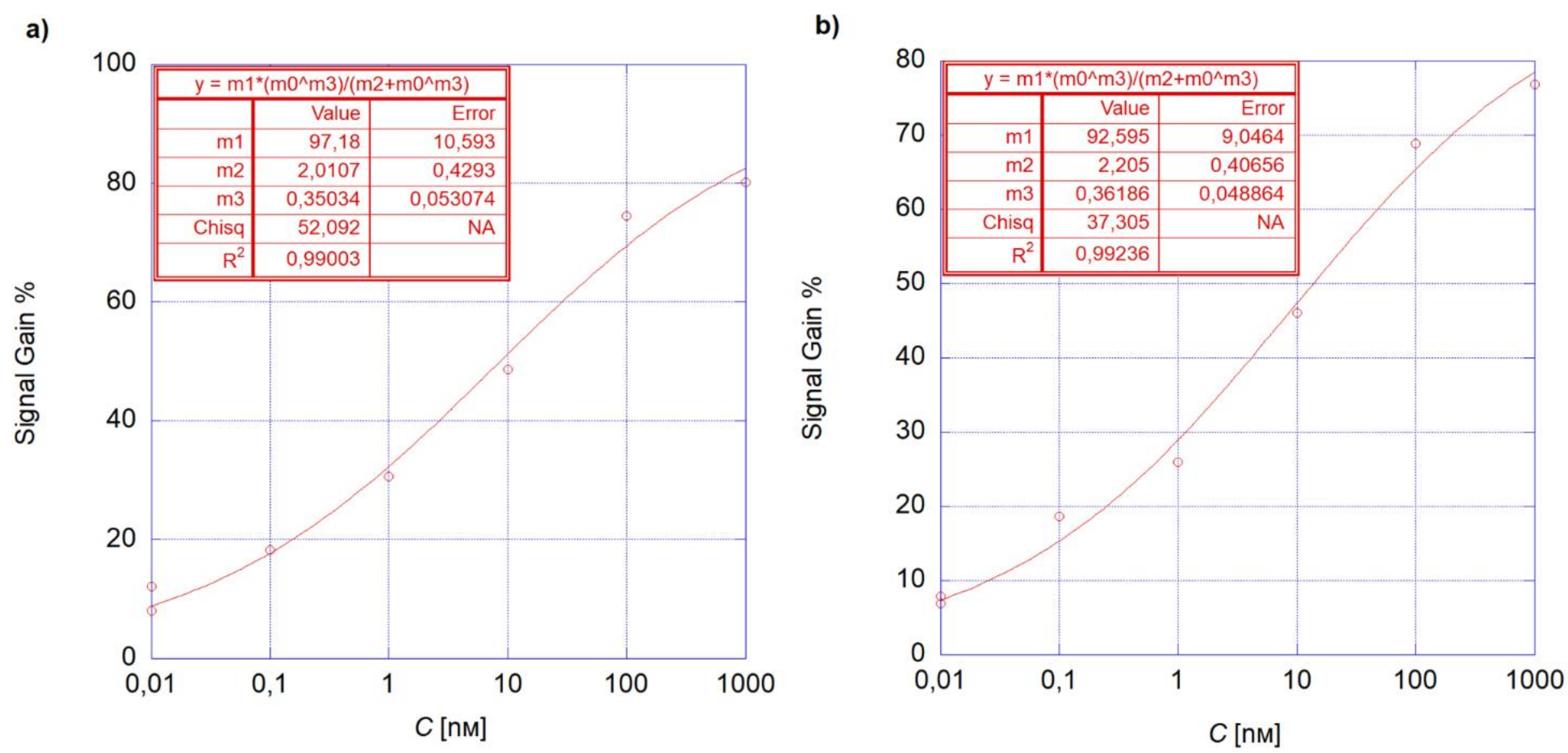

Figure S3. Langmuir-Hill isotherm fit of the miRNA-221sensor response to (a) DNA and (b) miRNA targets in buffer. Inset table show the estimated maximum signal gain (m_1) and dissociation constant (Kd, denoted as m_2).

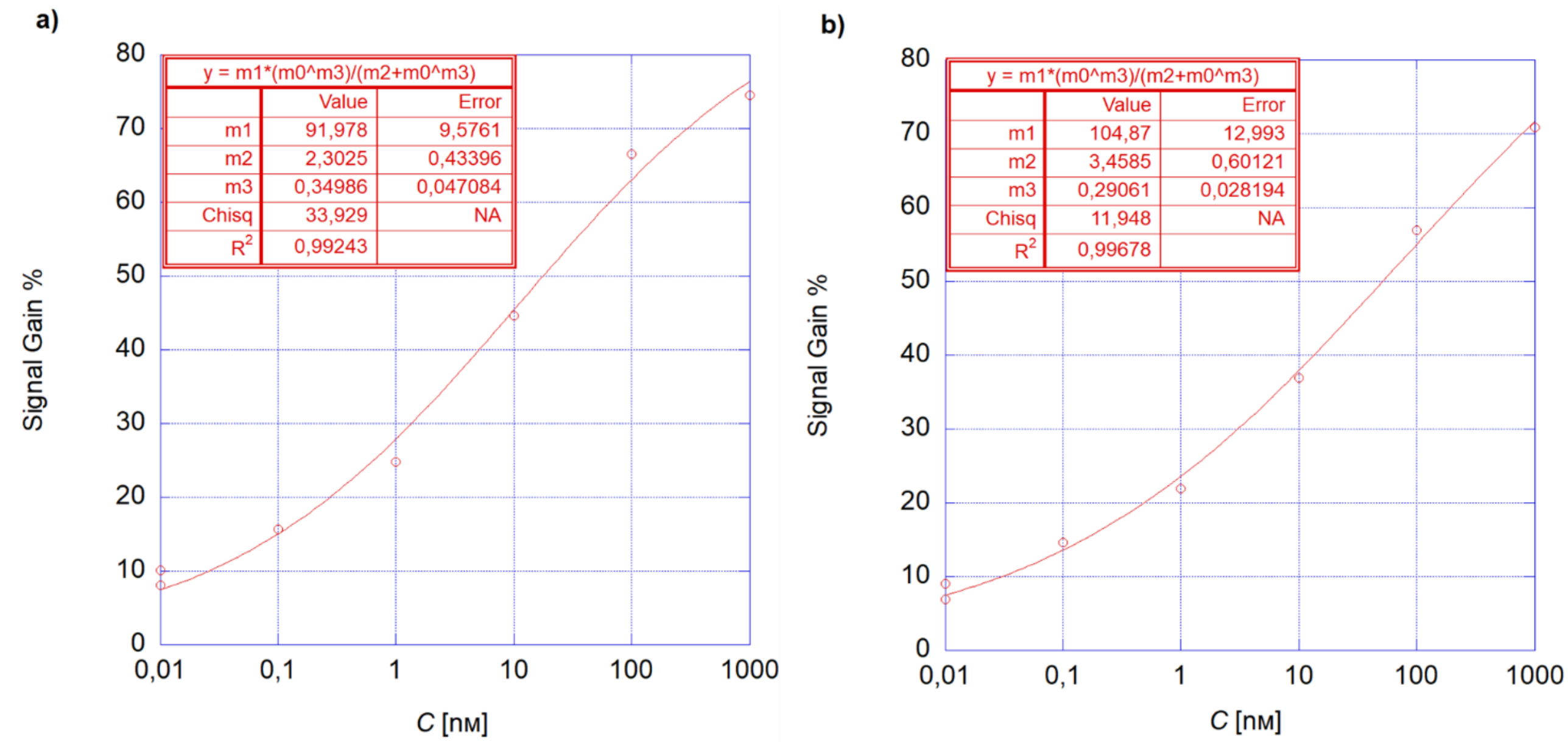

Figure S4. Langmuir-Hill isotherm fit of the miRNA-223 sensor response to (a) DNA and (b) miRNA targets in buffer. Inset table show the estimated maximum signal gain (m_1) and dissociation constant (Kd, denoted as m_2).

4. Oligonucleotide sequences

Table S4: Oligonucleotide sequences used in the development of E-DNA sensors.

Target	Type	Nucleotide sequence from 5' to 3'
miRNA-146b	Capture probe	$SH(CH_2)_6$-ACAGCCTATGGAATTCAGTTCTCA-MB
	ssDNA target	TGAGAACTGAATTCCATAGGCTGT
	miRNA target	UGAGAACUGAAUUCCAUAGGCUGU
	2bpmm DNA sequence	TGA**T**AACTGAATTCCATAG**T**CTGT
miRNA-221	Capture probe	$SH(CH_2)_6$-AAACCCAGCAGACAATGTAGCT-MB
	ssDNA target	AGCTACATTGTCTGCTGGGTTT
	miRNA target	AGCUACAUUGUCUGCUGGGUUU
	2bpmm DNA sequence	AG**A**TACATTGTCTGCTG**T**GTTT
miRNA-223	Capture probe	$SH(CH_2)_6$-TGGGGTATTTGACAAACTGACA-MB
	ssDNA target	TGTCAGTTTGTCAAATACCCCA
	miRNA target	UGUCAGUUUGUCAAAUACCCCA
	2bpmm DNA sequence	TGTCA**T**TTTGTCAAATAC**A**CCA
Control	Capture probe	$SH(CH_2)_6$-TAACCGATTTCAAATGGTGCTA-MB

References

[1] Wu, Yao, Farshad Tehrani, and Hazhir Teymourian, *et al.* "Microneedle Aptamer-Based Sensors for Continuous, Real-Time Therapeutic Drug Monitoring." *Analytical Chemistry* 94, no. 23 (2022): 8335–8345. https://doi.org/10.1021/acs.analchem.2c00829.
